# Tooth loss, denture use, and all-cause and cause-specific mortality in older adults: a community cohort study

**DOI:** 10.3389/fpubh.2023.1194054

**Published:** 2023-06-05

**Authors:** Miao Dai, Quhong Song, Taiping Lin, Xiaohong Huang, Yufang Xie, Xiang Wang, Liwei Zheng, Jirong Yue

**Affiliations:** ^1^Department of Geriatrics, Jiujiang First People’s Hospital, Jiujiang, Jiangxi, China; ^2^Department of Geriatrics and National Clinical Research Center for Geriatrics, West China Hospital of Sichuan University, Chengdu, Sichuan, China; ^3^Department of Cardiology, Jiujiang First People’s Hospital, Jiujiang, Jiangxi, China; ^4^National Clinical Research Center for Oral Diseases, West China Hospital for Stomatology, Sichuan University, Chengdu, Sichuan, China

**Keywords:** dental health, tooth loss, denture use, all-cause and cause-specific mortality, older adults

## Abstract

**Objectives:**

The available evidence on the connections between tooth loss, denture use, and mortality from all causes or specific causes among older adults is inconclusive. Therefore, we aimed to investigate the association between tooth loss, denture use, and all-cause and cause-specific mortality in older adults.

**Methods:**

A cohort of 5,403 participants aged 65 and older were recruited in the 2014 Chinese Longitudinal Healthy Longevity Survey wave and followed up in the 2018 wave. Cox proportional hazard models were used to examine the association between the number of natural teeth, denture use, and all-cause and cause-specific mortality.

**Results:**

During a mean (SD) follow-up of 3.1  years (1.3), 2,126 deaths (39.3%) occurred. Individuals with 0 and 1–9 teeth had higher mortality due to all-cause, cardiovascular disease (CVD), cancer, and other causes (all *p*-trend <0.05) than those with 20+ teeth. At the same time, no association was found with respiratory disease mortality. Participants who used dentures had lower mortality due to all causes [hazard ratios (HR) 0.79, 95% confidence intervals (CI) 0.71–0.88], CVD (HR 0.80, 95% CI 0.64–1.00), respiratory disease (HR 0.66, 95% CI 0.48–0.92), and other causes (HR 0.77, 95% CI 0.68–0.88) than those without dentures. Joint analysis revealed that older adults with fewer natural teeth and no dentures had higher mortality. Additionally, interaction analyses showed that the effects of the number of natural teeth on all-cause mortality were more pronounced in older adults aged <80  years (*p*-value for interaction = 0.03).

**Conclusion:**

Having fewer natural teeth, particularly less than 10 teeth, is linked to an increased risk of mortality from all causes, including CVD, cancer, and other causes, but not respiratory disease. The use of dentures would mitigate the adverse impact of tooth loss on all-cause and some cause-specific mortality.

## Introduction

Older adults represent the world’s fastest-growing demographic, with the population aged 60 years and up expected to reach 2.1 billion by 2050, up from 900 million in 2015 (1). Unfortunately, aging is associated with a higher likelihood of experiencing chronic diseases, disabilities, social isolation, and other age-related health issues, resulting in increased mortality rates (2). Therefore, understanding the risk factors associated with mortality among older people is vital to develop effective interventions and improve their health and well-being.

Dental health is fundamental to overall health and well-being, particularly in older adults. Natural tooth loss is a crucial factor affecting dental health in older adults. Research has linked tooth loss to several systemic health conditions, including cardiovascular disease (3), respiratory disease (4), diabetes (5), and cancer (6), which may increase the risk of death. The underlying mechanisms may be due to functional limitations (7), poor nutrition (8), and an increase in the total body inflammatory load (9). Despite growing evidence of an association between tooth loss and mortality in older adults, the findings are inconsistent. Some studies have found a strong association between tooth loss and increased mortality (10–14), while others have found no significant association (15–18). Denture use is a standard solution for tooth loss and has been shown to improve chewing ability and dietary intake (19). However, a few population-based studies have focused on the association between tooth loss and denture use and all-cause mortality (14, 20), with no attention to cause-specific mortality. For example, a prospective study found that periodontal disease and tooth loss were linked to elevated all-cause mortality and mortality caused by cardiovascular disease, respiratory disease, and endocrine/metabolic diseases among women aged 35–74 years (10). However, the study only looked at whether or not tooth loss had occurred and did not explore whether the severity of tooth loss or the use of dentures affected mortality. Additionally, only two studies have investigated the potential effect of dietary intake on the number of natural teeth and all-cause mortality (14, 20). Given the potential impact of diet on the relationship between oral health and mortality, it remains unclear whether the findings of previous studies can be extrapolated. Therefore, additional prospective studies of these populations are necessary to clarify the association between tooth loss, denture use, and all-cause and cause-specific mortality.

This study investigates the association between tooth loss, denture use, and all-cause and cause-specific mortality in older adults using data from the Chinese Longitudinal Healthy Longevity Survey (CLHLS) and explores their potential interaction. We hypothesize that tooth loss, whether partial or total, is associated with increased all-cause and cause-specific mortality in older adults and that using dentures may mitigate this adverse impact.

## Method

### Study design and participants

The CLHLS is an ongoing, prospective cohort study investigating the determinants of health and longevity among older Chinese adults. The study includes participants from 22 of China’s 31 provinces. The sample was selected using a multistage, stratified cluster sampling method, randomly choosing 631 cities and counties with the largest Han Chinese population. These sample sites represent approximately 85% of the Chinese population (21). The study commenced in 1998, with follow-up interviews conducted every 2–4 years. New participants were enrolled during the follow-up to minimize attrition due to death and loss of follow-up. All included individuals were interviewed face-to-face about determinants of health, including family relationships, socioeconomic characteristics, mental health, physical capacity, chronic disease, and other lifestyle-related measurements. A detailed account of the study design and methods is available (22).

Since the data on cause-specific deaths were only available in the 2018 wave, we used the baseline data from the 2014 wave of CLHLS and conducted a follow-up during the 2018 wave, with a response rate of 79.0%. During the 2014 wave, the study expanded to 23 provinces, adding Chengmai City in Hainan Province (Supplementary Figure S1). The 2014 wave included 7,192 participants. The exclusion criteria in the present study were: age <65 years (*n* = 85), those who were lost to follow-up (*n* = 1,511) or had incorrect death dates (*n* = 6), and missing data on the number of natural teeth and/or denture use (*n* = 187). Finally, 5,403 participants were included to analyze the association between the number of natural teeth, denture use, and all-cause and cause-specific mortality in older adults. A detailed description of the inclusion and exclusion process is shown in Supplementary Figure S2. We compared the baseline characteristics of the overall participants and the included participants in the 2014 wave (Supplementary Table S1). We found that the included participants more likely resided in rural areas, were economically dependent, and reported a lower rate of heart disease.

### Exposure assessment

The number of natural teeth was assessed by asking participants about the number of natural teeth they had at baseline. Participants were categorized into four groups based on their number of natural teeth: 0, 1–9, 10–19, and ≥20. Denture use was assessed by asking participants whether they had false teeth at baseline. Participants were categorized into two groups based on their denture use: with dentures and without dentures.

### Outcome

The cause of death was determined based on the International Classification of Diseases, 10th Revision (ICD-10) codes. The primary outcome was all-cause mortality. Cause-specific mortality was also examined for the following categories: cardiovascular disease (CVD) (codes I00-I99), respiratory disease (codes J00-99), cancer (codes C00-C97), and other causes. Mortality data were obtained from the participant’s next of kin or local doctors. Survival time was calculated from the first interview to the date of death or the last follow-up.

### Covariates

We controlled the potential confounding variables associated with the number of natural teeth, denture use, and mortality. These include age (continuous), sex, education (0 years, 1–6 years, or >6 years), residence (rural area or urban area), marital status [married or other (divorced, widowed, or never married)], living with family members [living with family member(s) or others (living alone or in an institution)], economic status (independence or dependence), smoking (never, current, or former), drinking (never, current, or former), regular exercise (never, current, or former), fruit intake (never, occasionally, or almost daily), vegetable intake (never, occasionally, or almost daily), meat intake (never, occasionally, or almost daily), fish intake (never, occasionally, or almost daily), and egg intake (never, occasionally, or almost daily), body mass index (BMI), chronic complications included self-reported hypertension (yes or no), heart disease (yes or no), diabetes mellitus (yes or no), respiratory disease (including bronchitis, emphysema, pneumonia) (yes or no), and cancer (yes or no). In addition, we classified BMI as underweight (<18.5 kg/m^2^), normal (18.5–23.9 kg/m^2^), overweight (24–27.9 kg/m^2^), and obese (≥28 kg/m^2^) (23). Supplementary Figure S3 shows the possible associations between variables.

### Statistical analysis

Missing data would reduce statistical power and biased estimates of the relationship. Therefore, we adopted a multiple imputation method based on the chain equation and five repetitions methods to solve the missing data (ranging from 0.2% to 8.3%) (Supplementary Table S2). Baseline characteristics of the study are presented as percentages for categorical variables and mean and standard deviations (SDs) for continuous variables.

Kaplan–Meier survival analysis was utilized to construct survival curves for the number of natural teeth and the denture use, with log-rank testing assessing differences between the groups. Cox proportional hazards models were used to estimate hazard ratios (HRs) and 95% confidence intervals (CIs) for all-cause and cause-specific mortality associated with the number of natural teeth (as a categorical or a continuous variable) and denture use. Model 1 was adjusted for baseline age, sex, residence, living arrangement, economic status, education, and marital status. Model 2 was further adjusted for smoking status, drinking status, regular exercise, fruit intake, vegetable intake, meat intake, fish intake, and egg intake, and further adjusted for denture use in the natural tooth model and further adjusted for the number of natural teeth in the denture use model. Model 3 was further adjusted for BMI, hypertension, heart disease, diabetes mellitus, respiratory disease, and cancer. To examine the individual impacts of tooth loss and denture use on mortality, we adjusted for denture use in the natural tooth model and the number of natural teeth in the denture use model. The crude incidence rate (IR) (per 1,000 person-years) of all-cause and cause-specific mortality was estimated. Linear trend tests were performed using the median values of varying categories of natural teeth numbers as a continuous variable. We used restricted cubic spline curves to examine the association of continuous values of natural teeth (20 natural teeth as reference) with all-cause and specific-cause mortality, with four knots at the 5th, 35th, 65th, and 95th percentiles of change in the natural teeth distribution. We conducted subgroup and interaction analyses by age, gender, denture use, residence, economic status, and BMI to investigate potential effect-modifying effects.

Furthermore, we conducted sensitivity analyses to examine the robustness of our main findings. First, we analyzed complete cases to evaluate the potential effects of the multiple imputation method. Second, to mitigate the potential influence of short-term follow-up, we excluded deaths within the first year, as the impact of oral health on mortality is often a chronic process. Third, we excluded participants with major chronic diseases at baseline, such as heart disease, respiratory disease, and cancer, to minimize some potential reverse causality. Finally, we used inverse probability weighting (IPW) based on the propensity scores to adjust for potential selection bias (24). The weights were the inverse of the probability of follow-up versus loss to follow-up, given the baseline covariates obtained *via* binary logistic regression. To assess the effectiveness of our propensity score modeling, we calculated a standardized mean difference (SMD) (all SMD <0.10).

All analyses were performed using R software version 4.1.3 (R Foundation for Statistical Computing), with a two-tailed *p* < 0.05 as the significance threshold.

## Results

### Basic characteristics of participants

A total of 5,403 older adults were included. The participants had a mean age of 85.4 years, with 53.8% being female. Table 1 presents the characteristics of the study population. Of all the participants, 80.6% reported having experienced tooth loss, and 35.7% of these individuals had lost all their teeth. The overall rate of denture usage was 35.1%, which increased proportionally with the number of teeth lost. Participants with fewer natural teeth tended to be older, female, unmarried, living alone, residing in rural areas, illiterate, economically dependent, using dentures, and underweight. Moreover, they were less likely to smoke, consume alcohol, exercise regularly, and suffer from hypertension, heart disease, diabetes mellitus, and respiratory diseases. They were likelier to have insufficient vegetables, fruits, meat, and fish intake.

**Table 1:** Characteristics of participants by the number of natural teeth and denture use.

Characteristics	Total (*n* = 5,403)	Number of natural teeth					Denture use		
		0 (*n* = 1,929)	1–9 (*n* = 1,518)	10–19 (*n* = 908)	20+ (*n* = 1,048)	*p*-value	Yes (*n* = 1,896)	No (*n* = 3,507)	*p*-value
Age (year), mean (SD)	85.44 (10.47)	89.93 (9.87)	87.00 (9.89)	81.63 (9.07)	78.21 (8.20)	<0.001	83.22 (9.42)	86.64 (10.81)	<0.001
Male, no. (%)	2,496 (46.2)	765 (39.7)	646 (42.6)	466 (51.3)	619 (59.1)	<0.001	942 (49.7)	1,554 (44.3)	<0.001
Married, no. (%)	2,184 (40.4)	537 (27.8)	538 (35.4)	456 (50.2)	653 (62.3)	<0.001	923 (48.7)	1,261 (36.0)	<0.001
Living with family member(s), no. (%)	4,232 (78.3)	1,505 (78.0)	1,150 (75.8)	718 (79.1)	859 (82.0)	0.002	1,501 (79.2)	2,731 (77.9)	0.29
Urban area, no. (%)	2,309 (42.7)	789 (40.9)	630 (41.5)	404 (44.5)	486 (46.4)	0.02	919 (48.5)	1,390 (39.6)	<0.001
Smoking status, no. (%)						<0.001			
Never	3,858 (71.4)	1,437 (74.5)	1,123 (74.0)	628 (69.2)	670 (63.9)		1,287 (67.9)	2,571 (73.3)	<0.001
Current	850 (15.7)	257 (13.3)	226 (14.9)	149 (16.4)	218 (20.8)		332 (17.5)	518 (14.8)	
Former	695 (12.9)	235 (12.2)	169 (11.1)	131 (14.4)	160 (15.3)		277 (14.6)	418 (11.9)	
Drinking status, no. (%)						<0.001			0.04
Never	4,071 (75.3)	1,514 (78.5)	1,163 (76.6)	676 (74.4)	718 (68.5)		1,391 (73.4)	2,680 (76.4)	
Current	807 (14.9)	248 (12.9)	206 (13.6)	141 (15.5)	212 (20.2)		311 (16.4)	496 (14.1)	
Former	525 (9.7)	167 (8.7)	149 (9.8)	91 (10.0)	118 (11.3)		194 (10.2)	331 (9.4)	
Regular exercise, no. (%)						<0.001			<0.001
Never	3,770 (69.8)	1,460 (75.7)	1,075 (70.8)	592 (65.2)	643 (61.4)		1,203 (63.4)	2,567 (73.2)	
Current	1,387 (25.7)	382 (19.8)	359 (23.6)	274 (30.2)	372 (35.5)		610 (32.2)	777 (22.2)	
Former	246 (4.6)	87 (4.5)	84 (5.5)	42 (4.6)	33 (3.1)		83 (4.4)	163 (4.6)	
Education (year), no. (%)						<0.001			<0.001
0	3,103 (57.4)	1,266 (65.6)	972 (64.0)	462 (50.9)	403 (38.5)		914 (48.2)	2,189 (62.4)	
1–6	1,731 (32.0)	522 (27.1)	438 (28.9)	338 (37.2)	433 (41.3)		717 (37.8)	1,014 (28.9)	
>6	569 (10.5)	141 (7.3)	108 (7.1)	108 (11.9)	212 (20.2)		265 (14.0)	304 (8.7)	
Economic independence, no. (%)	1,345 (24.9)	300 (15.6)	317 (20.9)	302 (33.3)	426 (40.6)	<0.001	571 (30.1)	774 (22.1)	<0.001
Number of natural teeth, no. (%)									<0.001
0	NA	NA	NA	NA	NA	NA	1,004 (53.0)	925 (26.4)	
1–9	NA	NA	NA	NA	NA		441 (23.3)	1,077 (30.7)	
10–19	NA	NA	NA	NA	NA		268 (14.1)	640 (18.2)	
20+	NA	NA	NA	NA	NA		183 (9.7)	865 (24.7)	
Denture use, no. (%)	1,896 (35.1)	1,004 (52.0)	441 (29.1)	268 (29.5)	183 (17.5)	<0.001	NA	NA	NA
BMI (kg/m^2^), no. (%)						<0.001			<0.001
Underweight (<18.5)	1,048 (19.4)	454 (23.5)	326 (21.5)	141 (15.5)	127 (12.1)		283 (14.9)	765 (21.8)	
Normal (18.5–24)	3,024 (56.0)	1,087 (56.4)	853 (56.2)	531 (58.5)	553 (52.8)		1,085 (57.2)	1,939 (55.3)	
Overweight (24–28)	1,023 (18.9)	302 (15.7)	267 (17.6)	182 (20.0)	272 (26.0)		409 (21.6)	614 (17.5)	
Obese (≥28)	308 (5.7)	86 (4.5)	72 (4.7)	54 (5.9)	96 (9.2)		119 (6.3)	189 (5.4)	
Hypertension, no. (%)	1,758 (32.5)	509 (26.4)	500 (32.9)	342 (37.7)	407 (38.8)	<0.001	653 (34.4)	1,105 (31.5)	0.03
Diabetes mellitus, no. (%)	281 (5.2)	74 (3.8)	65 (4.3)	64 (7.0)	78 (7.4)	<0.001	120 (6.3)	161 (4.6)	0.01
Heart disease, no. (%)	665 (12.3)	185 (9.6)	195 (12.8)	124 (13.7)	161 (15.4)	<0.001	260 (13.7)	405 (11.5)	0.02
Respiratory disease, no. (%)	591 (10.9)	184 (9.5)	187 (12.3)	89 (9.8)	131 (12.5)	0.01	228 (12.0)	363 (10.4)	0.07
Cancer, no. (%)	44 (0.8)	13 (0.7)	13 (0.9)	13 (1.4)	5 (0.5)	0.10	20 (1.1)	24 (0.7)	0.20
Fruit intake, no. (%)						<0.001			<0.001
Never	1,360 (25.2)	508 (26.3)	424 (27.9)	201 (22.1)	227 (21.7)		406 (21.4)	954 (27.2)	
Occasionally	1,863 (34.5)	639 (33.1)	549 (36.2)	337 (37.1)	338 (32.3)		597 (31.5)	1,266 (36.1)	
Almost daily	2,180 (40.3)	782 (40.5)	545 (35.9)	370 (40.7)	483 (46.1)		893 (47.1)	1,287 (36.7)	
Vegetables intake, no. (%)						<0.001			<0.001
Never	196 (3.6)	114 (5.9)	55 (3.6)	14 (1.5)	13 (1.2)		51 (2.7)	145 (4.1)	
Occasionally	452 (8.4)	191 (9.9)	148 (9.7)	61 (6.7)	52 (5.0)		125 (6.6)	327 (9.3)	
Almost daily	4,755 (88.0)	1,624 (84.2)	1,315 (86.6)	833 (91.7)	983 (93.8)		1,720 (90.7)	3,035 (86.5)	
Meat intake, no. (%)						<0.001			0.27
Never	365 (6.8)	165 (8.6)	106 (7.0)	41 (4.5)	53 (5.1)		124 (6.5)	241 (6.9)	
Occasionally	2,993 (55.4)	1,025 (53.1)	855 (56.3)	534 (58.8)	579 (55.2)		1,027 (54.2)	1,966 (56.1)	
Almost daily	2,045 (37.8)	739 (38.3)	557 (36.7)	333 (36.7)	416 (39.7)		745 (39.3)	1,300 (37.1)	
Fish intake, no. (%)						<0.001			0.001
Never	915 (16.9)	369 (19.1)	285 (18.8)	140 (15.4)	121 (11.5)		302 (15.9)	613 (17.5)	
Occasionally	4,072 (75.4)	1,424 (73.8)	1,119 (73.7)	684 (75.3)	845 (80.6)		1,415 (74.6)	2,657 (75.8)	
Almost daily	416 (7.7)	136 (7.1)	114 (7.5)	84 (9.3)	82 (7.8)		179 (9.4)	237 (6.8)	
Egg intake, no. (%)						0.10			<0.001
Never	488 (9.0)	182 (9.4)	148 (9.7)	66 (7.3)	92 (8.8)		169 (8.9)	319 (9.1)	
Occasionally	3,310 (61.3)	1,141 (59.1)	928 (61.1)	584 (64.3)	657 (62.7)		1,090 (57.5)	2,220 (63.3)	
Almost daily	1,605 (29.7)	606 (31.4)	442 (29.1)	258 (28.4)	299 (28.5)		637 (33.6)	968 (27.6)	

Values are presented as number (%) or mean ± SD. Differences in characteristics were compared using the *χ*^2^ test for categorical variables and ANOVA or *t*-test for continuous variables. BMI, body mass index.

### Association of the number of natural teeth with all-cause and cause-specific mortality

**Figure 1:**
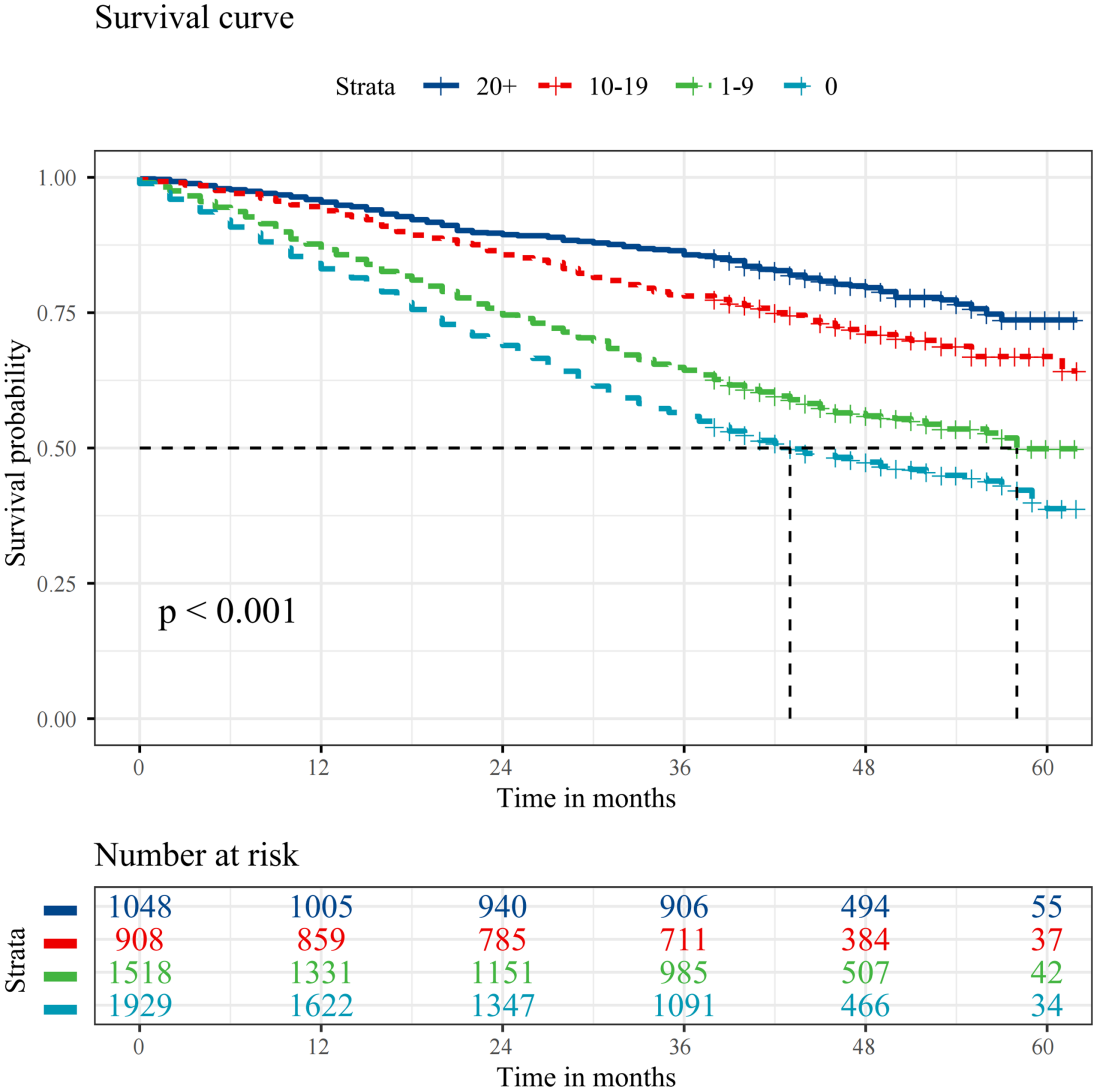
Kaplan–Meier survival curves for all-cause mortality according to the number of natural teeth. The median survival duration is represented using a vertical dashed line.

Over a median follow-up of 3.4 years (interquartile range: 2.3 to 4.1, 16794.3 person-years), 2,126 (39.3%) participants died, with 435 (20.5%) deaths attributed to CVD, 216 (10.2%) to respiratory diseases, 107 (5.0%) to cancer, and 1,368 (64.3%) to other causes. Kaplan–Meier curves indicate that individuals with fewer teeth have a significantly lower chance of survival (Figure 1). Specifically, participants with 0 teeth had the lowest probability of survival, followed by those with 1–9 teeth, 10–19 teeth, and those with 20 or more natural teeth (log-rank test: *p* < 0.001) (Figure 1).

Individuals with 0 teeth and 1–9 teeth exhibited a substantially higher risk of all-cause mortality (0 teeth: HR 1.66, 95% CI 1.41–1.96; 1–9 teeth: HR 1.43, 95% CI 1.21–1.68) than those with 20+ teeth (Table 2). In addition, cause-specific analyses reveal that individuals with 0 and 1–9 natural teeth had significantly higher mortality from CVD (0 teeth: HR 1.83, 95% CI 1.28–2.63; 1–9 teeth: HR 1.57, 95% CI 1.10–2.24), cancer (0 teeth: HR 2.21, 95% CI 1.13–4.33; 1–9 teeth: HR 2.11, 95% CI 1.13–3.92), and other causes (0 teeth: HR 1.71, 95% CI 1.38–2.12; 1–9 teeth: HR 1.42, 95% CI 1.15–1.76) than those with 20+ teeth, but not from respiratory disease (Table 2). Furthermore, the hazard ratios (HRs) for the risk of death for the number of teeth (continuous variable) were as follows: 0.98 (0.97–0.98) for all-cause mortality, 0.98 (0.96–0.99) for cardiovascular disease (CVD) mortality, 0.97 (0.95–0.99) for cancer mortality, and 0.98 (0.97–0.98) for mortality from other causes (Table 2).

**Table 2:** Hazard ratios for all-cause and cause-specific mortality according to the number of natural teeth or denture use.

Characteristic	Number of deaths (incidence rate, per 1,000 person-year, %)	Unadjusted model	Model 1	Model 2	Model 3
		HR (95% CI)	HR (95% CI)	HR (95% CI)	HR (95% CI)
Number of natural teeth					
All-cause mortality					
20+	214 (56.7)	Reference	Reference	Reference	Reference
10–19	257 (82.4)	1.45 (1.21–1.74)	1.11 (0.92–1.33)	1.12 (0.93–1.34)	1.11 (0.92–1.34)
1–9	657 (143.4)	2.54 (2.18–2.96)	1.39 (1.19–1.64)	1.43 (1.21–1.68)	1.43 (1.21–1.68)
0	998 (187.6)	3.33 (2.87–3.86)	1.54 (1.32–1.80)	1.65 (1.40–1.95)	1.66 (1.41–1.96)
*p*-value for trend^a^		<0.001	<0.001	<0.001	<0.001
Number of teeth (continuous variable)		0.95 (0.95–0.96)	0.98 (0.98–0.99)	0.98 (0.97–0.99)	0.98 (0.97–0.98)
Cardiovascular disease mortality					
20+	45 (13.2)	Reference	Reference	Reference	Reference
10–19	60 (22.1)	1.67 (1.13–2.45)	1.23 (0.83–1.82)	1.26 (0.85–1.86)	1.32 (0.89–1.95)
1–9	139 (38.2)	2.86 (2.04–4.00)	1.44 (1.02–2.05)	1.49 (1.04–2.12)	1.57 (1.10–2.24)
0	191 (49.0)	3.64 (2.63–5.04)	1.56 (1.11–2.19)	1.67 (1.16–2.39)	1.83 (1.28–2.63)
*p*-value for trend^a^		<0.001	0.007	0.004	<0.001
Number of teeth (continuous variable)		0.95 (0.94–0.96)	0.98 (0.97–0.99)	0.98 (0.97–0.99)	0.98 (0.96–0.99)
Respiratory disease mortality					
20+	30 (8.9)	Reference	Reference	Reference	Reference
10–19	33 (12.5)	1.40 (0.85–2.29)	1.07 (0.65–1.77)	1.02 (0.62–1.70)	1.03 (0.62–1.73)
1–9	62 (17.8)	1.99 (1.28–3.07)	1.23 (0.78–1.93)	1.27 (0.80–2.01)	1.25 (0.79–1.99)
0	91 (24.3)	2.70 (1.78–4.07)	1.43 (0.93–2.21)	1.57 (0.99–2.50)	1.67 (0.98–2.65)
*p*-value for trend^a^		<0.001	0.08	0.04	0.06
Number of teeth (continuous variable)		0.96 (0.95–0.98)	0.99 (0.97–1.00)	0.98 (0.97–1.00)	0.98 (0.96–1.00)
Cancer mortality					
20+	17 (5.1)	Reference	Reference	Reference	Reference
10–19	20 (7.6)	1.50 (0.79–2.87)	1.56 (0.81–3.00)	1.62 (0.83–3.13)	1.55 (0.79–3.04)
1–9	34 (9.9)	1.95 (1.09–3.48)	2.12 (1.16–3.87)	2.11 (1.13–3.91)	2.11 (1.13–3.92)
0	36 (9.8)	1.94 (1.09–3.46)	2.12 (1.15–3.89)	2.11 (1.08–4.11)	2.21 (1.13–4.33)
*p*-value for trend^a^		0.01	0.007	0.01	0.01
Number of teeth (continuous variable)		0.98 (0.96–1.00)	0.97 (0.95–0.99)	0.97 (0.95–0.99)	0.97 (0.95–0.99)
Other cause mortality					
20+	122 (34.1)	Reference	Reference	Reference	Reference
10–19	144 (49.8)	1.46 (1.15–1.86)	1.03 (0.81–1.31)	1.05 (0.82–1.34)	1.03 (0.81–1.32)
1–9	422 (101.4)	2.98 (2.43–3.64)	1.39 (1.13–1.72)	1.42 (1.15–1.76)	1.42 (1.15–1.76)
0	680 (142.3)	4.17 (3.44–5.06)	1.61 (1.31–1.97)	1.72 (1.39–2.13)	1.71 (1.38–2.12)
*p*-value for trend^a^		<0.001	<0.001	<0.001	<0.001
Number of teeth (continuous variable)		0.94 (0.93–0.95)	0.98 (0.97–0.99)	0.98 (0.97–0.98)	0.98 (0.97–0.98)
Denture use					
All-cause mortality					
Without dentures	1,523 (144.8)	Reference	Reference	Reference	Reference
With dentures	603 (96.1)	0.66 (0.60–0.73)	0.88 (0.80–0.97)	0.79 (0.71–0.87)	0.79 (0.71–0.88)
Cardiovascular disease mortality					
Without dentures	304 (36.6)	Reference	Reference	Reference	Reference
With dentures	131 (24.5)	0.67 (0.55–0.82)	0.90 (0.73–1.11)	0.81 (0.64–1.01)	0.80 (0.64–1.00)
Respiratory disease mortality					
Without dentures	154 (19.1)	Reference	Reference	Reference	Reference
With dentures	62 (12.0)	0.63 (0.47–0.84)	0.79 (0.58–1.06)	0.71 (0.51–0.98)	0.66 (0.48–0.92)
Cancer mortality					
Without dentures	61 (7.7)	Reference	Reference	Reference	Reference
With dentures	46 (8.9)	1.15 (0.78–1.68)	1.17 (0.79–1.73)	0.92 (0.60–1.43)	0.90 (0.58–1.40)
Other cause mortality					
Without dentures	957 (100.5)	Reference	Reference	Reference	Reference
With dentures	333 (57.9)	0.60 (0.53–0.68)	0.86 (0.76–0.98)	0.76 (0.67–0.87)	0.77 (0.68–0.88)

HR, hazard ratio; CI, confidence interval. Model 1: adjusted for baseline age, sex, residence, living arrangement, economic status, education, and marital status. Model 2: further adjusted for smoking status, drinking status, regular exercise, fruit intake, vegetable intake, meat intake, fish intake, and egg intake, and further adjusted for denture use in the natural tooth model and further adjusted for the number of natural teeth in the denture use model. Model 3: further adjusted for body mass index, hypertension, heart disease, diabetes mellitus, respiratory disease, and cancer.

a Test for trend based on the variable containing the median value for each group.

**Figure 2:**
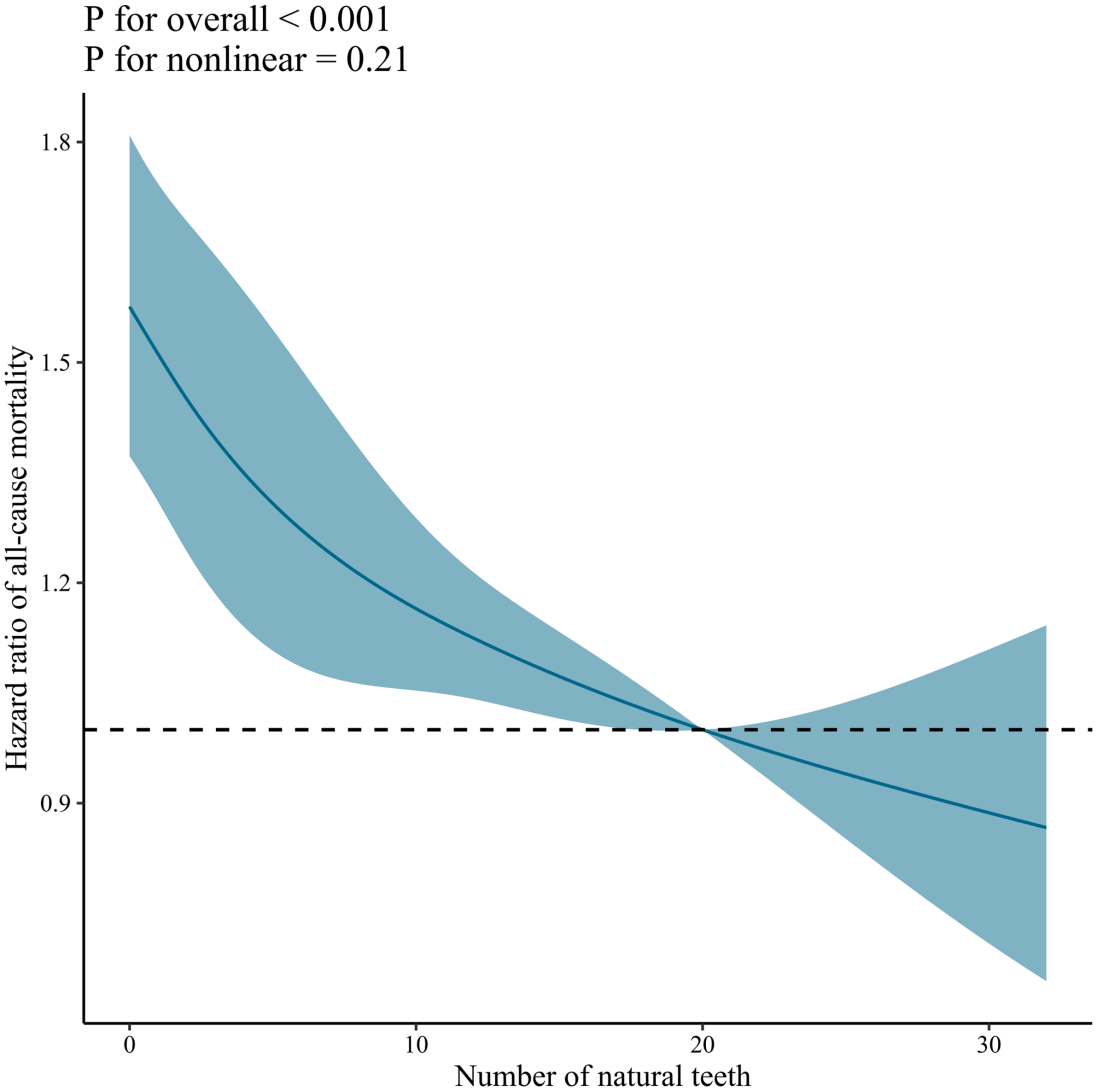
Dose–response association between the number of natural teeth and all-cause mortality. Solid blue lines are multivariable-adjusted hazard ratios, with shaded areas showing 95% confidence intervals derived from restricted cubic spline regressions with four knots at the 5th, 35th, 65th, and 95th percentiles. The reference was set at 20 natural teeth. Multivariate models were adjusted for baseline age, sex, marital status, education, residence, living arrangement, economic status, smoking status, drinking status, regular exercise, denture use, body mass index, hypertension, heart disease, diabetes mellitus, respiratory disease, cancer, fruit intake, vegetable intake, meat intake, fish intake, and egg intake.

Restricted cubic spline analysis shows a linear trend in the correlation between the number of natural teeth and mortality due to all-cause, CVD, respiratory disease, and other causes (Figure 2; Supplementary Figure S4). However, no linear or non-linear relationship was found between the number of natural teeth and cancer mortality (Supplementary Figure S4).

### Association of denture use with all-cause and cause-specific mortality

The Kaplan–Meier analyses of denture use indicate that participants with dentures had higher survival than those without (Supplementary Figure S5). In addition, individuals with dentures demonstrated a lower risk of all-cause mortality (HR 0.79, 95% CI 0.71–0.88), CVD mortality (HR 0.80, 95%CI 0.64–1.00), respiratory disease mortality (HR 0.66, 95% CI 0.48–0.92), and mortality attributed to other causes (HR 0.77, 95% CI 0.68–0.88) than those without dentures. However, no significant difference was observed in cancer mortality (Table 2).

### Combined association of the number of natural teeth and denture use with all-cause and cause-specific mortality

The Kaplan–Meier survival curves stratified by the number of natural teeth and denture use show that individuals with 0 teeth without dentures exhibited the lowest survival rates, whereas those with 10–19 teeth with dentures had the highest survival rates (Supplementary Figure S6). In addition, individuals with 0 teeth without dentures, 0 teeth with dentures, and 1–9 teeth without dentures had a significantly higher risk of all-cause mortality, CVD mortality, and other cause mortality than those with 20+ teeth with/without dentures (Table 3). Specifically, the HRs for all-cause mortality were 1.71, 1.37, and 1.48. The corresponding HRs for CVD mortality were 1.85, 1.55, and 1.65. The HRs for other causes of mortality were 1.76, 1.38, and 1.45. Moreover, individuals who had 0 teeth without dentures were also at a significantly higher risk of respiratory disease mortality (HR 1.75, 95% CI 1.06–2.87) than those who had 20+ teeth with/without dentures, while those who had 0 teeth with dentures and 1–9 teeth without dentures had a significantly higher risk of cancer mortality (HR 2.27, 95% CI 1.20–4.29 and HR 2.35, 95% CI 1.20–4.57, respectively) (Table 3). In contrast, individuals with 10–19 teeth with/without dentures and 1–9 teeth with dentures were not found to have a significantly high risk of all-cause mortality or cause-specific mortality when compared to those with 20+ teeth with/without dentures (Table 3).

**Table 3:** Combined effects of the number of natural teeth and denture use on all-cause and cause-specific mortality.

Characteristic	Number of deaths (Incidence rate, per 1,000 person-year, %)	Unadjusted model	Model 1	Model 2	Model 3
		HR (95% CI)	HR (95% CI)	HR (95% CI)	HR (95% CI)
All-cause mortality					
20+	214 (56.7)	Reference	Reference	Reference	Reference
10–19 with dentures	51 (51.9)	0.91 (0.67–1.24)	0.88 (0.65–1.19)	0.87 (0.64–1.19)	0.87 (0.64–1.18)
10–19 without dentures	206 (96.4)	1.71 (1.41–2.07)	1.21 (1.00–1.47)	1.17 (0.96–1.42)	1.16 (0.96–1.41)
1–9 with dentures	122 (80.6)	1.43 (1.14–1.78)	1.21 (0.97–1.51)	1.18 (0.94–1.48)	1.17 (0.93–1.46)
1–9 without dentures	535 (174.4)	3.10 (2.65–3.64)	1.51 (1.28–1.79)	1.47 (1.24–1.74)	1.48 (1.25–1.75)
0 with dentures	402 (130.2)	2.31 (1.95–2.72)	1.39 (1.17–1.65)	1.35 (1.14–1.60)	1.37 (1.15–1.62)
0 without dentures	596 (266.9)	4.79 (4.09–5.60)	1.80 (1.52–2.14)	1.71 (1.44–2.03)	1.71 (1.44–2.03)
*p*-value for trend^a^		<0.001	<0.001	<0.001	<0.001
Cardiovascular disease mortality					
20+	45 (13.2)	Reference	Reference	Reference	Reference
10–19 with dentures	14 (15.6)	1.17 (0.64–2.14)	1.15 (0.63–2.10)	1.17 (0.64–2.14)	1.26 (0.69–2.30)
10–19 without dentures	46 (25.4)	1.91 (1.27–2.88)	1.28 (0.84–1.93)	1.25 (0.83–1.90)	1.30 (0.86–1.98)
1–9 with dentures	26 (19.6)	1.48 (0.91–2.40)	1.21 (0.75–1.97)	1.19 (0.73–1.93)	1.21 (0.74–1.97)
1–9 without dentures	113 (48.9)	3.64 (2.57–5.14)	1.56 (1.08–2.25)	1.55 (1.07–2.24)	1.65 (1.14–2.38)
0 with dentures	87 (34.9)	2.60 (1.81–3.73)	1.45 (1.00–2.10)	1.41 (0.98–2.05)	1.55 (1.07–2.25)
0 without dentures	104 (74.2)	5.48 (3.87–7.78)	1.75 (1.20–2.56)	1.69 (1.15–2.47)	1.85 (1.26–2.71)
*p*-value for trend^a^		<0.001	0.006	0.012	0.003
Respiratory disease mortality					
20+	30 (8.9)	Reference	Reference	Reference	Reference
10–19 with dentures	8 (9.1)	1.02 (0.47–2.23)	1.01 (0.46–2.21)	0.94 (0.43–2.06)	0.94 (0.42–2.07)
10–19 without dentures	25 (14.1)	1.59 (0.93–2.70)	1.13 (0.66–1.94)	1.01 (0.59–1.75)	1.02 (0.59–1.78)
1–9 with dentures	8 (6.2)	0.70 (0.32–1.52)	0.62 (0.28–1.35)	0.61 (0.28–1.33)	0.57 (0.26–1.25)
1–9 without dentures	54 (24.6)	2.74 (1.75–4.28)	1.55 (0.96–2.48)	1.46 (0.91–2.36)	1.47 (0.91–2.37)
0 with dentures	41 (17.1)	1.90 (1.19–3.05)	1.28 (0.79–2.08)	1.21 (0.74–1.96)	1.22 (0.75–1.98)
0 without dentures	50 (37.1)	4.10 (2.61–6.46)	1.80 (1.10–2.96)	1.63 (0.99–2.68)	1.75 (1.06–2.87)
*p*-value for trend^a^		<0.001	0.07	0.12	0.09
Cancer mortality					
20+	17 (5.1)	Reference	Reference	Reference	Reference
10–19 with dentures	6 (6.8)	1.35 (0.53–3.43)	1.36 (0.53–3.47)	1.32 (0.52–3.37)	1.14 (0.44–3.00)
10–19 without dentures	14 (8.0)	1.58 (0.78–3.21)	1.67 (0.82–3.44)	1.75 (0.85–3.59)	1.74 (0.84–3.59)
1–9 with dentures	11 (8.4)	1.66 (0.78–3.55)	1.76 (0.82–3.79)	1.71 (0.80–3.70)	1.64 (0.76–3.55)
1–9 without dentures	23 (10.8)	2.12 (1.13–3.96)	2.36 (1.22–4.59)	2.30 (1.18–4.49)	2.35 (1.20–4.57)
0 with dentures	26 (10.9)	2.15 (1.17–3.96)	2.30 (1.23–4.33)	2.17 (1.15–4.10)	2.27 (1.20–4.29)
0 without dentures	10 (7.9)	1.56 (0.71–3.40)	1.77 (0.76–4.10)	1.72 (0.74–4.00)	1.77 (0.76–4.11)
*p*-value for trend^a^		0.01	0.01	0.01	0.01
Other cause mortality					
20+	122 (34.1)	Reference	Reference	Reference	Reference
10–19 with dentures	23 (25.0)	0.73 (0.47–1.14)	0.68 (0.44–1.07)	0.67 (0.43–1.05)	0.66 (0.42–1.03)
10–19 without dentures	121 (61.4)	1.80 (1.40–2.32)	1.17 (0.91–1.51)	1.14 (0.88–1.47)	1.13 (0.87–1.45)
1–9 with dentures	77 (53.8)	1.58 (1.19–2.10)	1.27 (0.95–1.69)	1.22 (0.91–1.62)	1.2 (0.90–1.60)
1–9 without dentures	345 (126.4)	3.72 (3.03–4.58)	1.50 (1.21–1.87)	1.45 (1.17–1.81)	1.45 (1.17–1.81)
0 with dentures	248 (88.7)	2.60 (2.10–3.23)	1.41 (1.13–1.76)	1.35 (1.08–1.69)	1.38 (1.10–1.72)
0 without dentures	432 (218.1)	6.45 (5.27–7.89)	1.90 (1.53–2.37)	1.78 (1.43–2.22)	1.76 (1.41–2.20)
*p*-value for trend^a^		<0.001	<0.001	<0.001	<0.001

HR, hazard ratio; CI, confidence interval. Model 1: adjusted for baseline age, sex, residence, living arrangement, economic status, education, and marital status. Model 2: further adjusted for smoking status, drinking status, regular exercise, fruit intake, vegetable intake, meat intake, fish intake, and egg intake. Model 3: further adjusted for body mass index, hypertension, heart disease, diabetes mellitus, respiratory disease, and cancer.

a Test for trend based on the variable containing the median value for each group.

### Effect modification

Supplementary Figure S7 reveals that the association between the number of natural teeth and all-cause mortality was more pronounced in older adults aged <80 years (than those aged ≥80 years) (*p*-value for interaction = 0.03). Among individuals aged ≥80 years, only those with fewer than 10 teeth had a higher risk of all-cause mortality. No other variables, including sex, residence, BMI, economic status, and denture use, significantly modified the association between the number of natural teeth and denture use and all-cause and cause-specific mortality (Supplementary Figures S8–12).

### Sensitivity analysis

The associations between the number of natural teeth and denture use with all-cause and cause-specific mortality remained consistent after excluding participants with missing covariate values (Supplementary Table S3). Similarly, after removing participants who died within one year of follow-up, no essential changes in the results were observed (Supplementary Table S3). Additionally, no substantive changes in the results were observed when excluding participants with pre-existing conditions such as heart disease, respiratory disease, and cancer (Supplementary Table S3). Finally, after conducting IPW using propensity scores, no substantive differences were observed in the results (Supplementary Table S3).

## Discussion

This community-based cohort study in China adds to the existing literature on the association between tooth loss, the use of dentures, and mortality among older adults. Our findings reveal that participants with fewer natural teeth, particularly those with 0 and 1–9 teeth and those without dentures, are at a higher risk for all-cause and cause-specific mortality, particularly for CVD, cancer, and other causes. Moreover, this association persisted after adjusting for potential confounding factors, such as dietary intake, socioeconomic status, and other health behaviors. In addition, among participants without dentures, those with 0 teeth or 1–9 teeth had a higher mortality risk than those with 20+ teeth and with/without dentures. Conversely, dentures appeared to mitigate mortality in participants with partial tooth loss.

The findings of this study are consistent with previous prospective studies in which tooth loss was associated with a higher risk of all-cause mortality (11, 25–28). A cohort study found that older adults who experience tooth loss were at a higher risk of mortality, and the use of dentures was found to have a protective effect against all-cause mortality, regardless of the extent of tooth loss (14). A study of 50,045 people between the ages of 40 and 75 in northeastern Iran found that participants with the most severe tooth loss had a higher overall mortality rate than those who lost the fewest teeth (25). Our results and previous studies suggest that preserving as many natural teeth as possible and using dentures can reduce the risk of all-cause mortality. However, some studies have reported results that are inconsistent with our findings. For example, in a study of community-dwelling older adults in Japan, Maekawa et al. (28) found that functional teeth were a significant independent risk factor for mortality, whereas the number of present teeth was not. Additionally, two other studies reported no associations between the number of teeth and mortality among older men (16, 17). Possible explanations are differences in the target study participants’ age, geographic location, lifestyle, ethnicity, or cultural ecology. Unlike existing studies that are limited to a single geographical location or a small area, our study covered a large area of 23 research sites in 23 provinces in mainland China. In addition, different dietary patterns related to oral health in different study populations may affect the association between dental status and all-cause mortality.

Additionally, the relationship between tooth loss, denture use, and mortality associated with specific causes is noteworthy. In cause-specific analyses, participants with 0 and 1–9 teeth are at a higher risk for CVD, cancer, and other cause of mortality. Similarly, among participants without dentures, those with 0 teeth had a higher risk of respiratory disease mortality. This is consistent with previous studies showing a link between tooth loss and CVD mortality (26) and respiratory mortality (10). Our study adds to the literature by demonstrating that denture use may reduce cause-specific mortality among older adults with tooth loss. Specifically, our study found that participants with dentures in those with partial tooth loss had lower CVD mortality, respiratory disease mortality, and other cause mortality than those without dentures. Limited studies have investigated the relationship between denture use and cancer mortality. While some studies suggest that good oral health may reduce the risk of certain types of cancer (29), there is no evidence that denture use alone can reduce the risk of cancer mortality. The relationship between tooth loss, denture use, and cancer mortality is complex and may be influenced by factors such as cancer type and disease stage. Furthermore, the limited number of cancer-related deaths within our sample may have led to a wide confidence interval of our findings, indicating the necessity for further research into the relationship between tooth loss, denture use, and cancer, including the need to increase the sample size.

However, we found that the HRs for tooth loss were all relatively close to 1, suggesting that the associations between tooth loss and mortality may not be very strong. Notably, small effect sizes do not necessarily indicate that the relationship between tooth loss and mortality is not significant. In some cases, an HR closer to 1 may be meaningful and clinically relevant, particularly if the outcome being studied significantly impacts a participant’s health outcomes. Additionally, our study was conducted on a large population-based sample, enhancing our findings’ generalizability. The number of natural teeth may be a risk marker rather than a risk indicator of mortality. However, our study provides valuable insights into the potential health consequences of tooth loss in older adults, highlighting the importance of maintaining good oral health as we age.

The mechanism underlying the associations of tooth loss and denture use with all-cause and cause-specific mortality remains uncertain, but several possibilities have been proposed. First, natural tooth loss can affect chewing ability, reduce dietary diversity, and induce poor oral hygiene (30). This may result in reduced overall nutrient intake and can increase the risk of systemic inflammation (31) and chronic diseases such as CVD (32) and cancer (33). Our study found a significant association between tooth loss and insufficient intake of essential food groups at baseline, including vegetables, fruits, meat, and fish. However, it is unclear whether the diet is a confounding factor or a link between tooth loss and mortality. A poor diet could be a risk factor for increased mortality, but it could also be a consequence of tooth loss. While our study cannot differentiate between the two possibilities, it is important to consider both when interpreting the results. Therefore, further studies considering diet as a potential confounder or mediator are warranted. Furthermore, natural tooth loss can cause psychological distress and social isolation, contributing to poorer mental health and ultimately increased mortality (34). However, our study did not investigate the association between tooth loss with psychological distress and social isolation in older adults. Thus, further studies are required to verify the findings and elucidate the underlying mechanisms. Restorative treatments, on the other hand, such as denture use, are frequently used to improve chewing function and are thought to improve nutritional status (35, 36). Our baseline data showed that participants with dentures were more likely to consume adequate amounts of vegetables, fruits, meat, fish, and eggs than those without dentures. In terms of public health implications, ensuring that people who have lost teeth receive appropriate restorative treatment restores oral function. It improves swallowing function (37) and maintains protein intake, playing an essential role in maintaining the health of old adults, such as reducing mortality in old adults.

Tooth loss can result from various factors, such as tooth decay and gum disease (38). In addition, poor oral hygiene (39), smoking (40), and medical conditions, such as diabetes (41) and osteoporosis (42), may also contribute to tooth loss. Aging can also play a role, as the enamel on teeth gradually wears down over time. Another notable point in this study is that age may modify the relation between tooth loss and all-cause mortality with a stronger positive association in individuals aged<80 years. It may be that older adults aged <80 years may be more socially active and engaged in their communities (43), and tooth loss could impact their ability to communicate and interact with others (44). This social isolation could exacerbate the negative impact of tooth loss on their mental and emotional well-being. In contrast, older adults aged ≥80 years may have a higher level of resilience and adaptability to the adverse effects of tooth loss. They may have experienced tooth loss earlier in their lives and have found ways to cope with its impacts. Overall, the findings of our study suggest that age is an essential factor to consider when examining the association between tooth loss and mortality. It is important to further explore the mechanisms underlying this relationship and identify strategies to promote oral health and prevent tooth loss in older adults. However, our study was designed as an observational cohort study, with inherent limitations in establishing causality. Further research, such as randomized controlled trials, will establish a causal relationship between tooth loss and all-cause mortality.

### Strengths and limitations

This study provides a valuable contribution to the existing literature on the role of the number of natural teeth and dentures in predicting all-cause and cause-specific mortality in older adults. This study’s strengths include using a large sample of older adults, examining the number of natural teeth, and using dentures as separate measures of dental health. The number of natural teeth and the use of dentures provide different information about oral health status and are dependent and correlated variables. Therefore, separately analyzing them may allow for a more nuanced understanding of the relationship between oral health status and other variables of interest. It can also provide valuable information for researchers and policymakers in designing interventions and policies to improve oral health outcomes. However, this study has several limitations to consider. First, the study was based on the self-reported number of natural teeth and denture use, which may be subjected to reporting bias. Second, we could not assess the effect of changes in dental health over time, as dental health was only assessed at baseline. Third, we did not have information on the cause of tooth loss, which could affect the association between the number of natural teeth and mortality. Fourth, the study was limited to older adults in China, and the findings may not be generalizable to other populations. Finally, as with any analysis, residual confusion caused by unmeasured factors cannot be eliminated.

## Conclusion

In conclusion, our study indicates that older adults with a higher number of tooth loss and who do not use dentures are at increased mortality, particularly from all-cause, cardiovascular disease, cancer, and other causes. These findings indicate that maintaining adequate natural teeth is crucial in preventing mortality among older adults. They also highlight the potential benefits of denture use in mitigating the adverse effects of tooth loss on overall and cause-specific mortality.

## Data availability statement

Publicly available datasets were analyzed in this study. This data can be found at: https://opendata.pku.edu.cn/dataverse/CHADS.

## Ethics statement

The studies involving human participants were reviewed and approved by Ethics Committee of Peking University (IRB00001052-13074). The patients/participants provided their written informed consent to participate in this study.

## Author contributions

All authors contributed to the article and approved the submitted version.

## Funding

This research was funded by grants from the Science and Technology Plan of Jiangxi Provincial Health Commission (202310092), Chinese National Science & Technology Pillar Program (2020YFC2005600), 1.3.5 project for disciplines of excellence, West China Hospital, Sichuan University (ZYJC21005), and National Clinical Research Center for Geriatrics, West China Hospital, Sichuan University (Z20191012).

## Conflict of interest

The authors declare that the research was conducted in the absence of any commercial or financial relationships that could be construed as a potential conflict of interest.

## Publisher’s note

All claims expressed in this article are solely those of the authors and do not necessarily represent those of their affiliated organizations, or those of the publisher, the editors and the reviewers. Any product that may be evaluated in this article, or claim that may be made by its manufacturer, is not guaranteed or endorsed by the publisher.
