# Corrigendum: Tooth loss, denture use, and all-cause and cause-specific mortality in older adults: a community cohort study

**DOI:** 10.3389/fpubh.2023.1360927

**Published:** 2024-01-09

**Authors:** Miao Dai, Quhong Song, Taiping Lin, Xiaohong Huang, Yufang Xie, Xiang Wang, Liwei Zheng, Jirong Yue

**Affiliations:** ^1^Department of Geriatrics, Jiujiang First People's Hospital, Jiujiang, Jiangxi, China; ^2^Department of Geriatrics and National Clinical Research Center for Geriatrics, West China Hospital of Sichuan University, Chengdu, Sichuan, China; ^3^Department of Cardiology, Jiujiang First People's Hospital, Jiujiang, Jiangxi, China; ^4^National Clinical Research Center for Oral Diseases, West China Hospital for Stomatology, Sichuan University, Chengdu, Sichuan, China

**Keywords:** dental health, tooth loss, denture use, all-cause and cause-specific mortality, older adults

In the published article, there was an error in the Funding statement. The grant number “SKJP220225994” was corrected to “202310092”. The correct Funding statement appears below.

## Funding

This research was funded by grants from the Science and Technology Plan of Jiangxi Provincial Health Commission (202310092), Chinese National Science & Technology Pillar Program (2020YFC2005600), 1.3.5 project for disciplines of excellence, West China Hospital, Sichuan University (ZYJC21005), and National Clinical Research Center for Geriatrics, West China Hospital, Sichuan University (Z20191012).

The authors apologize for this error and state that this does not change the scientific conclusions of the article in any way. The original article has been updated.

