# Supplementary material for: Tooth loss, denture use, and all-cause and cause-specific mortality in older adults: a community cohort study
Source: Front Public Health. 2023 Jun 5;11:1194054. doi: 10.3389/fpubh.2023.1194054 (PMC10277727; doi:10.3389/fpubh.2023.1194054)
Supplement: Supplementary file 1 [file Table_1.pdf]

Supplementary Material

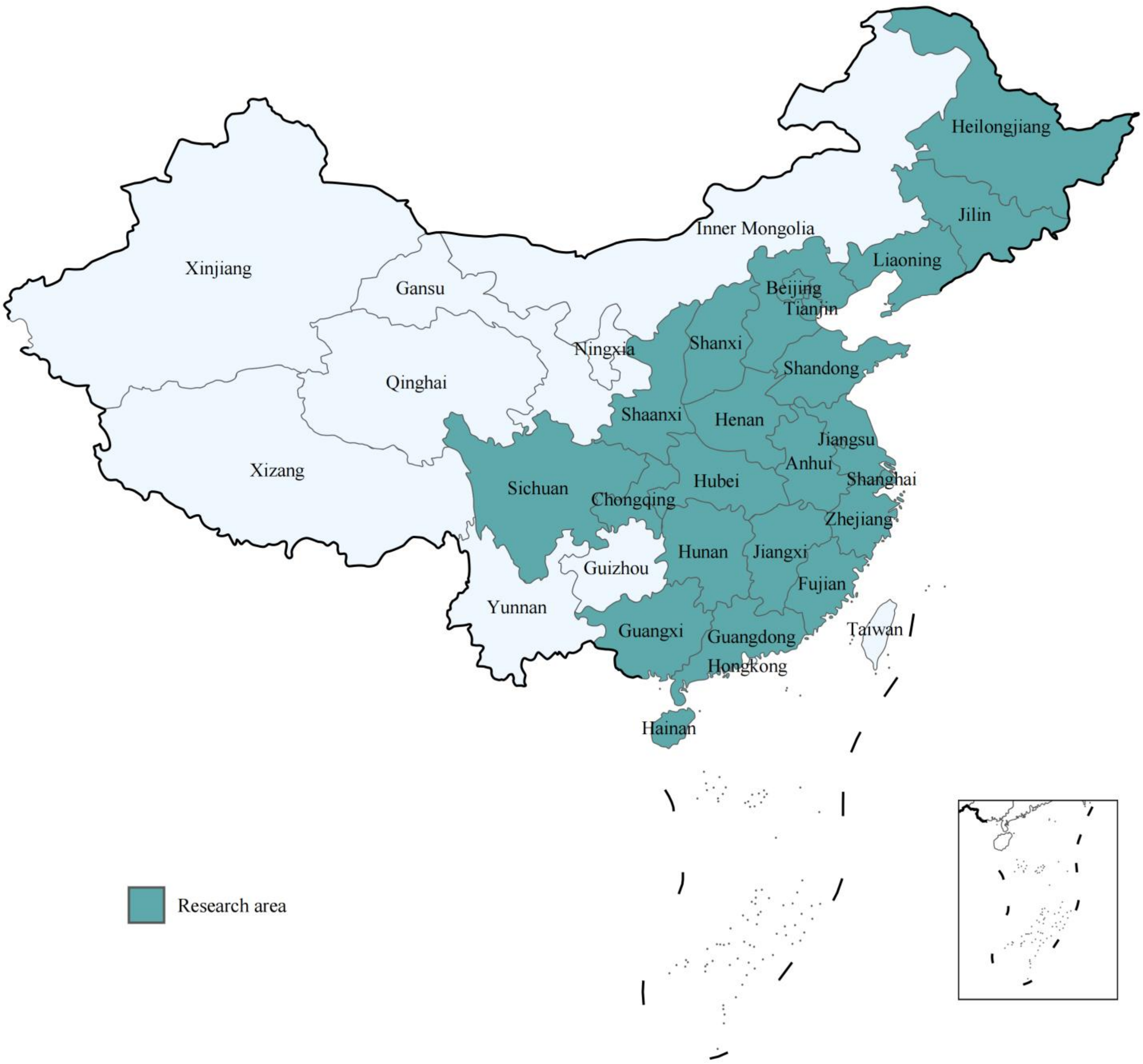

Figure S1 The 23 research areas included in the Chinese Longitudinal Healthy Longevity Survey (CLHLS) in mainland China in the 2014 wave

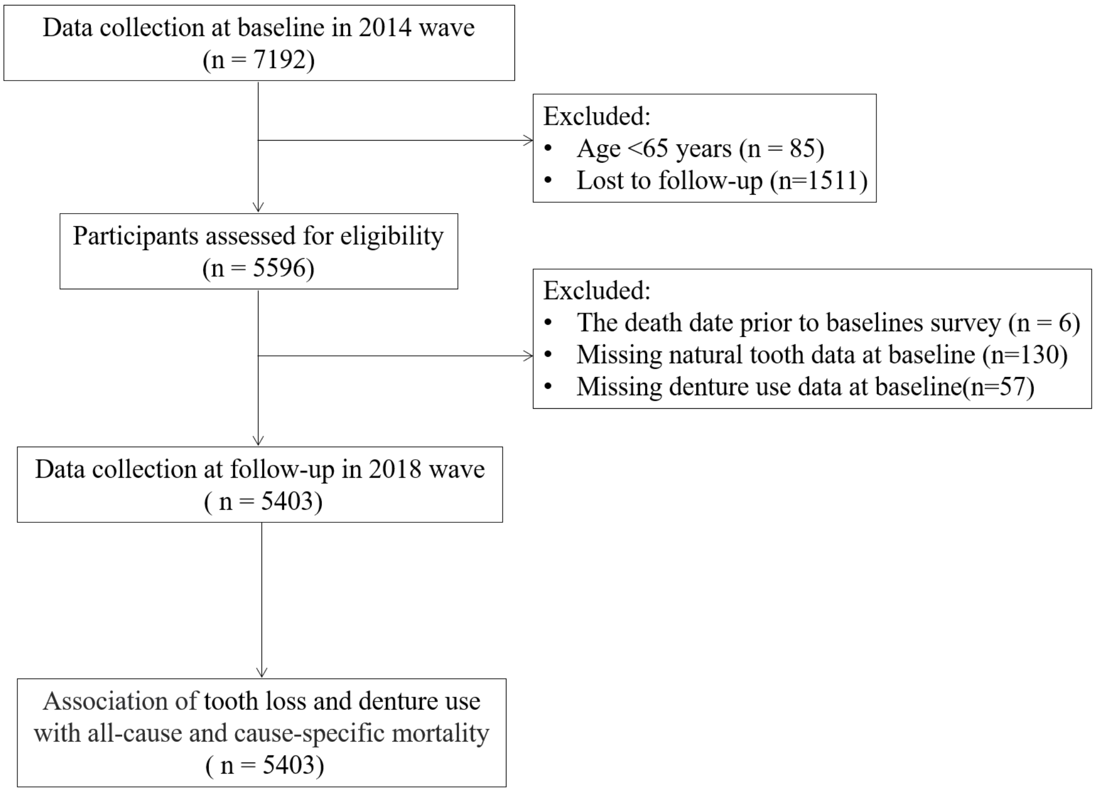

Figure S2 Flowchart of the included study population.

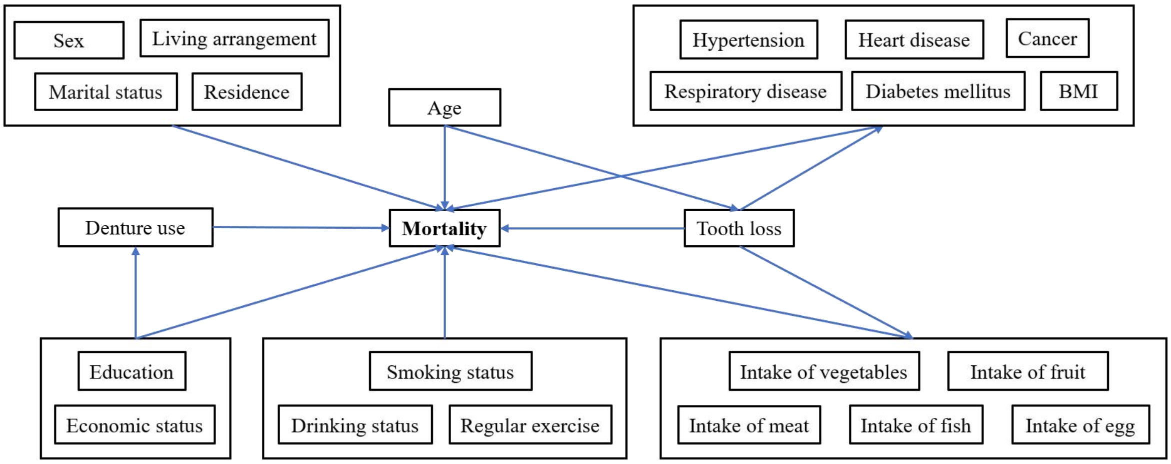

Figure S3 The acyclic graph of the possible associations between variables.

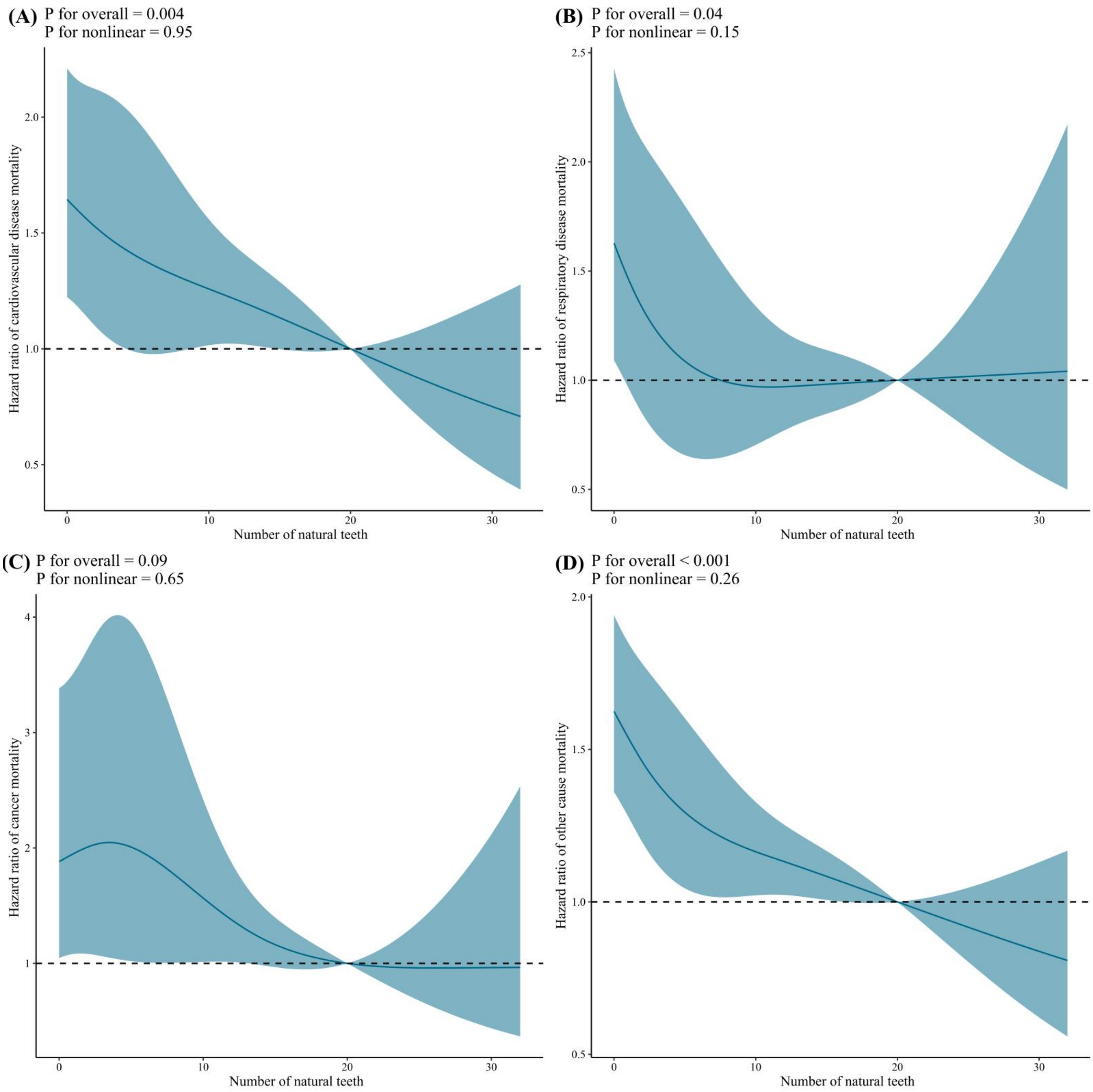

Figure S4 Dose-response association between the number of natural teeth and cause-specific mortality. Notes: Solid blue lines are multivariable-adjusted hazard ratios, with shaded areas showing 95% confidence intervals derived from restricted cubic spline regressions with four knots at the 5th, 35th, 65th, and 95th percentiles. The reference was set at 20 natural teeth. Multivariate models were adjusted for baseline age, sex, marital status, education, residence, living arrangement, economic status, smoking status, drinking status, regular exercise, body mass index, denture use, hypertension, heart disease, diabetes mellitus, respiratory disease, cancer, fruit intake, vegetable intake, meat intake, fish intake, and egg intake.

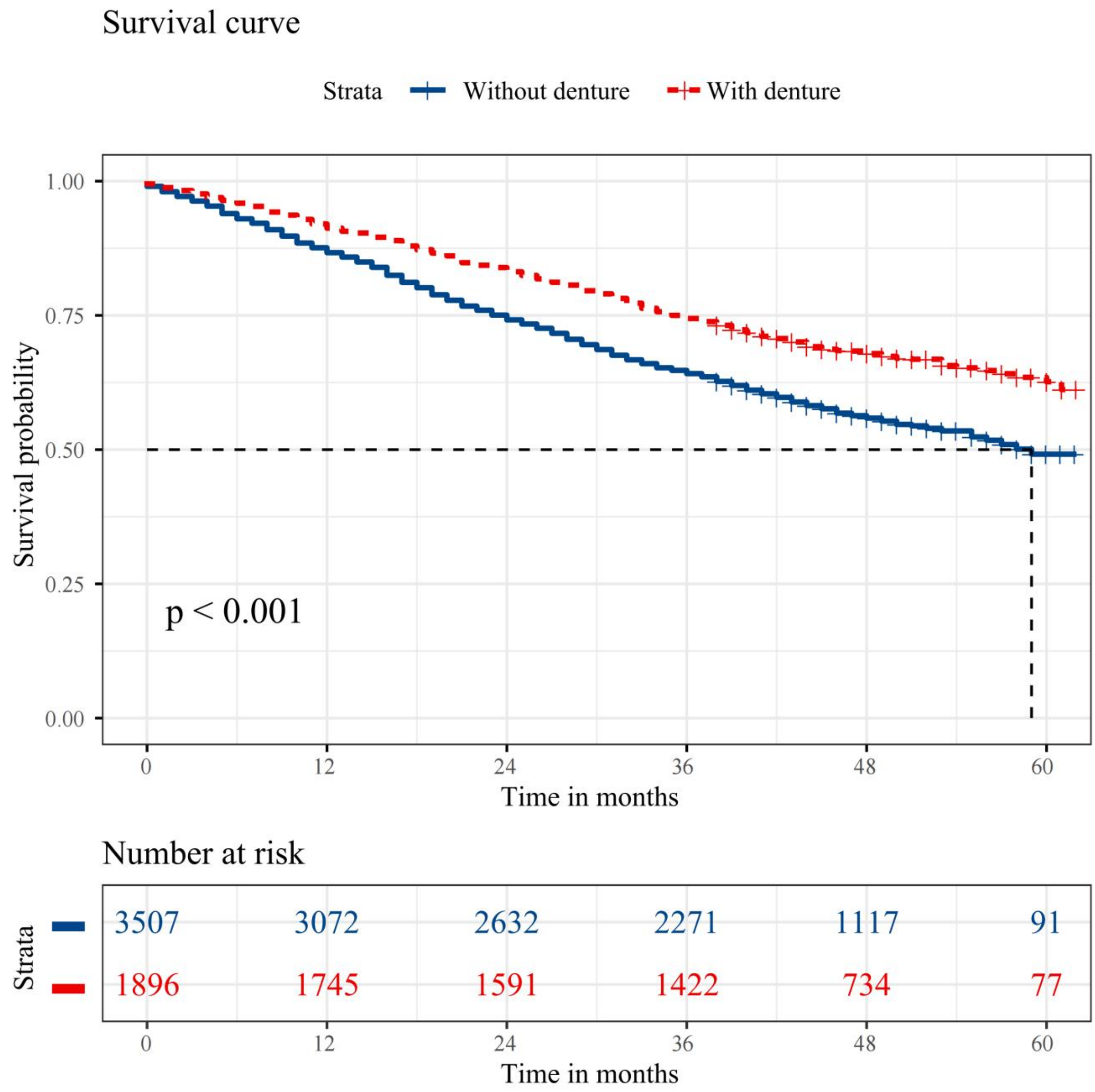

Figure S5 Kaplan-Meier survival curves for all-cause mortality according to denture use. The median survival duration is represented using a vertical dashed line.

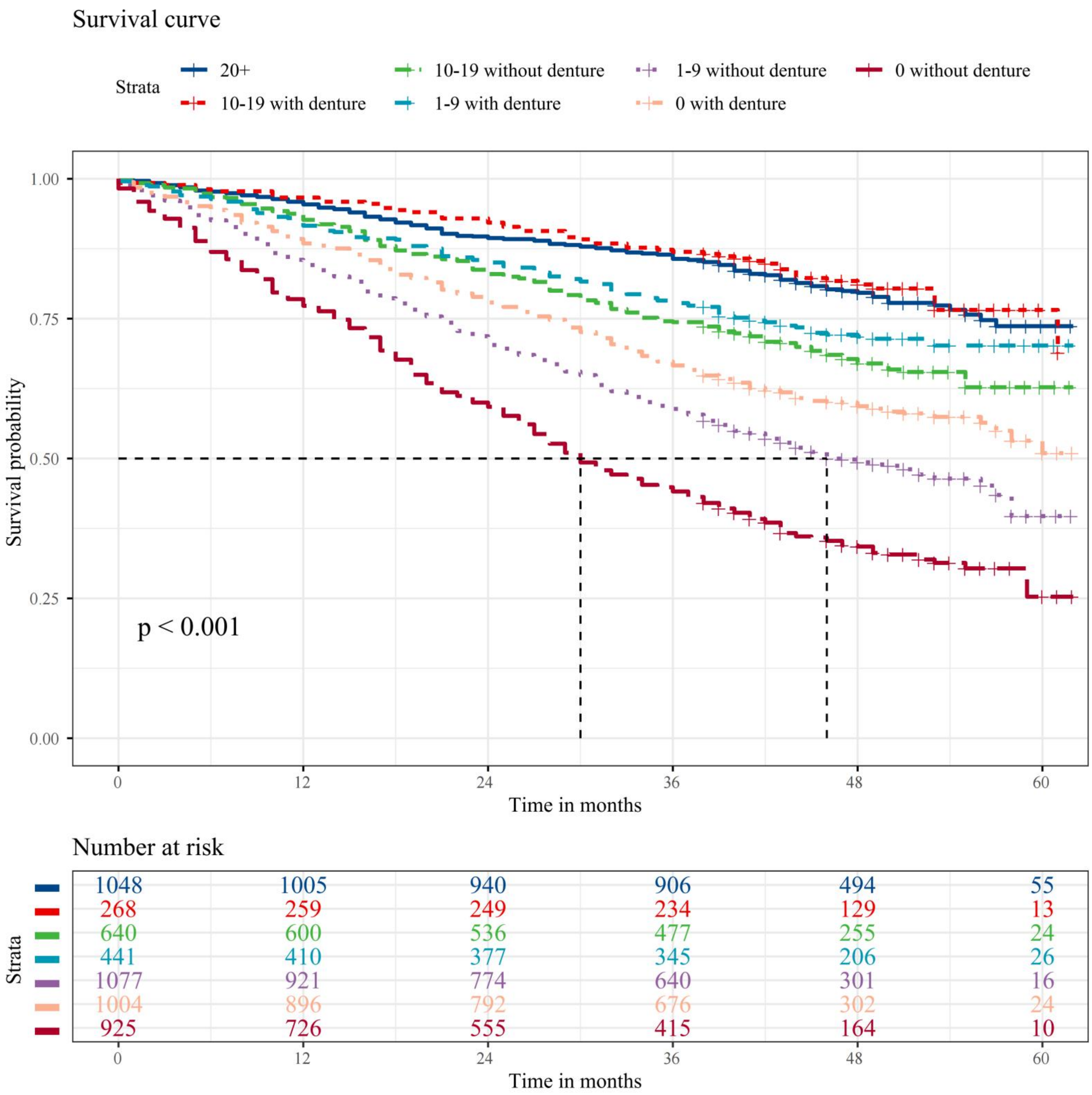

Figure S6 Kaplan-Meier survival curves for all-cause mortality according to the number of natural teeth and denture use. The median survival duration is represented using a vertical dashed line.

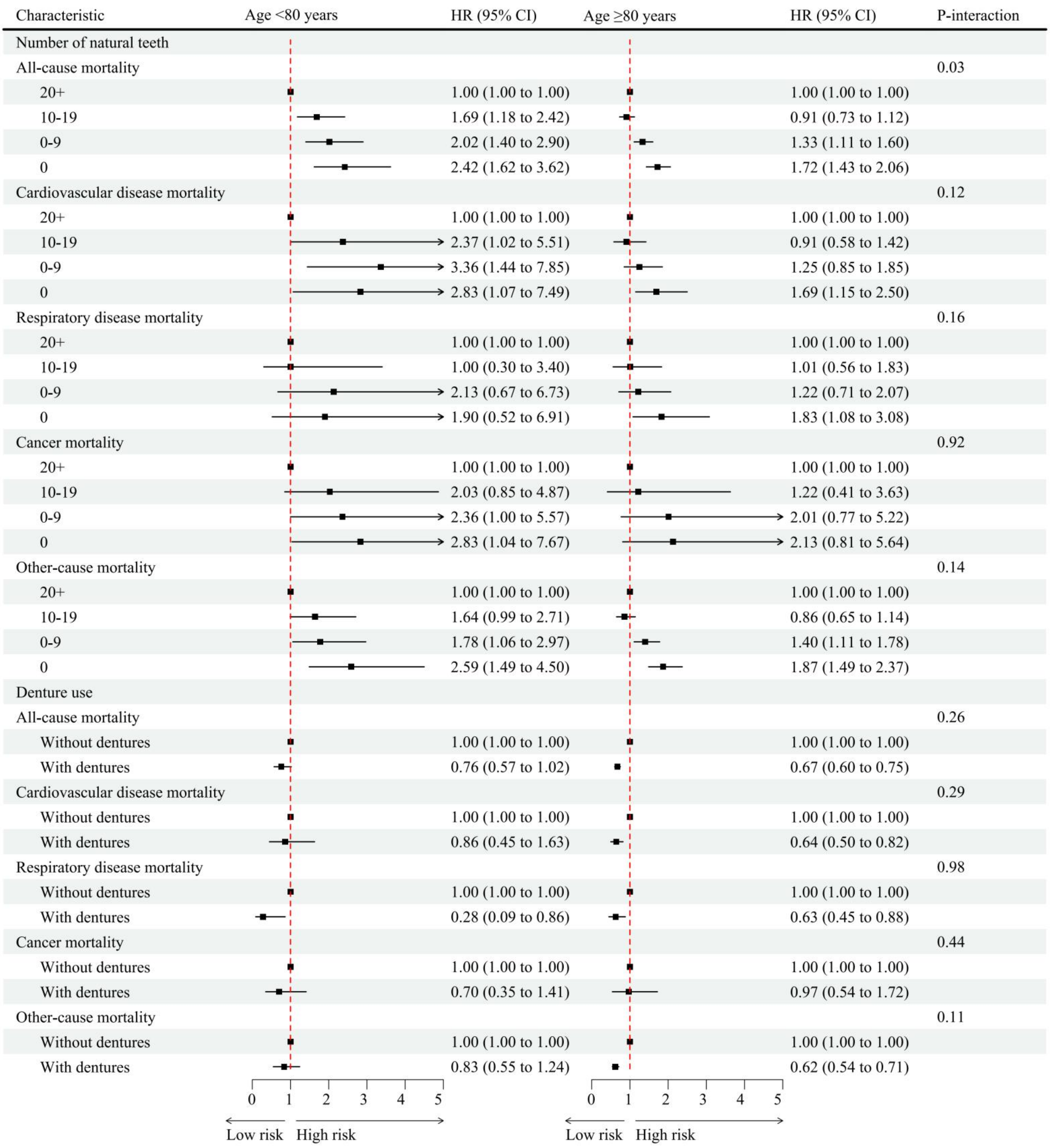

Figure S7 Association of the number of natural teeth and denture use with all-cause and cause-specific mortality stratified by baseline age. Multivariate models were adjusted for baseline sex, marital status, education, residence, living arrangement, economic status, smoking status, drinking status, regular exercise, body mass index, hypertension, heart disease, diabetes mellitus, respiratory disease, cancer, fruit intake, vegetable intake, meat intake, fish intake, and egg intake, and further adjusted for denture use in the natural tooth and further adjusted for the number of natural teeth in the denture use model. HR, hazard ratio; CI, confidence interval.

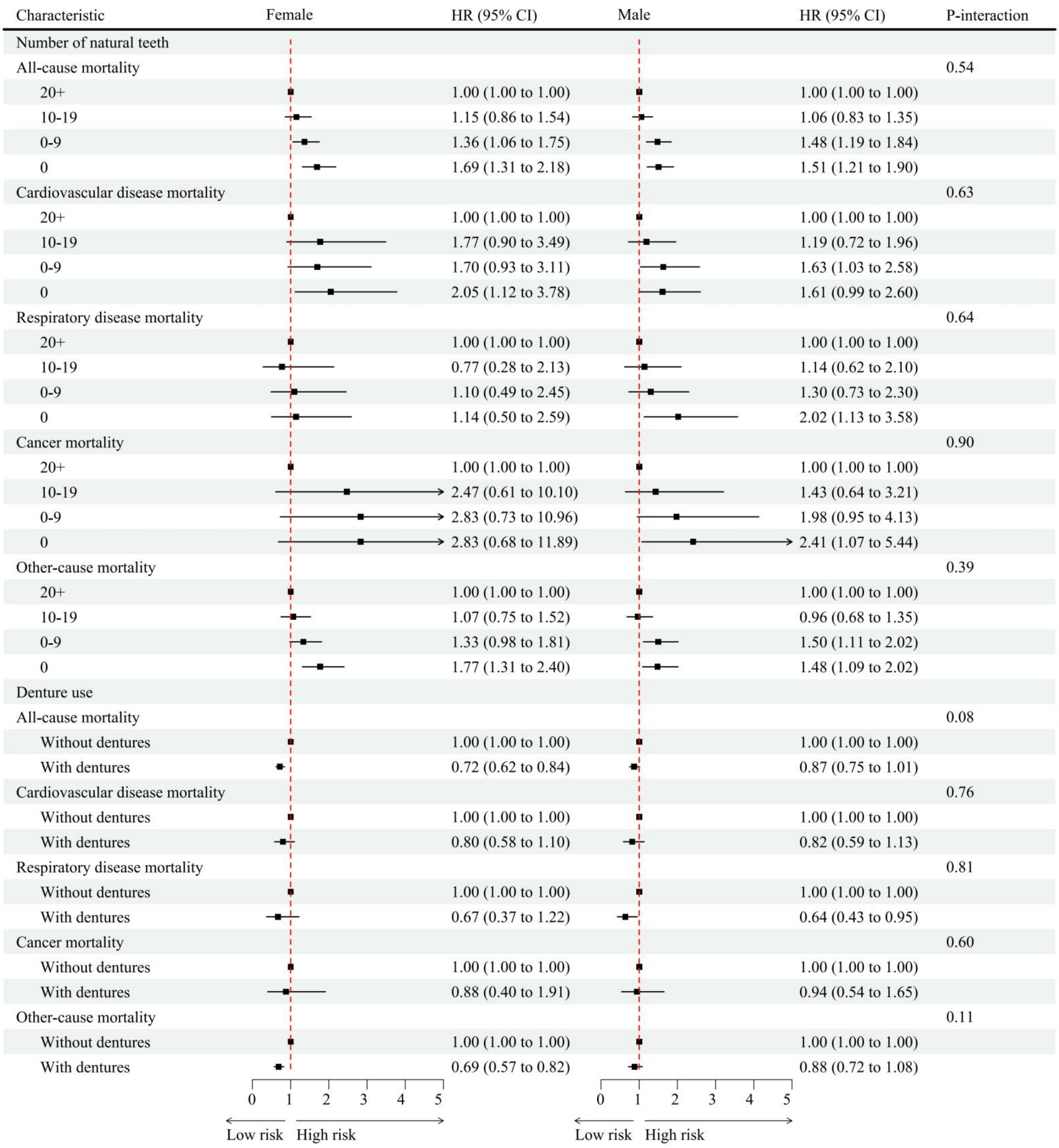

Characteristic	Female HR (95% CI)	Male HR (95% CI)	P-interaction
Number of natural teeth			
All-cause mortality			0.54
20+	1.00 (1.00 to 1.00)	1.00 (1.00 to 1.00)	
10-19	1.15 (0.86 to 1.54)	1.06 (0.83 to 1.35)	
0-9	1.36 (1.06 to 1.75)	1.48 (1.19 to 1.84)	
0	1.69 (1.31 to 2.18)	1.51 (1.21 to 1.90)	
Cardiovascular disease mortality			0.63
20+	1.00 (1.00 to 1.00)	1.00 (1.00 to 1.00)	
10-19	1.77 (0.90 to 3.49)	1.19 (0.72 to 1.96)	
0-9	1.70 (0.93 to 3.11)	1.63 (1.03 to 2.58)	
0	2.05 (1.12 to 3.78)	1.61 (0.99 to 2.60)	
Respiratory disease mortality			0.64
20+	1.00 (1.00 to 1.00)	1.00 (1.00 to 1.00)	
10-19	0.77 (0.28 to 2.13)	1.14 (0.62 to 2.10)	
0-9	1.10 (0.49 to 2.45)	1.30 (0.73 to 2.30)	
0	1.14 (0.50 to 2.59)	2.02 (1.13 to 3.58)	
Cancer mortality			0.90
20+	1.00 (1.00 to 1.00)	1.00 (1.00 to 1.00)	
10-19	2.47 (0.61 to 10.10)	1.43 (0.64 to 3.21)	
0-9	2.83 (0.73 to 10.96)	1.98 (0.95 to 4.13)	
0	2.83 (0.68 to 11.89)	2.41 (1.07 to 5.44)	
Other-cause mortality			0.39
20+	1.00 (1.00 to 1.00)	1.00 (1.00 to 1.00)	
10-19	1.07 (0.75 to 1.52)	0.96 (0.68 to 1.35)	
0-9	1.33 (0.98 to 1.81)	1.50 (1.11 to 2.02)	
0	1.77 (1.31 to 2.40)	1.48 (1.09 to 2.02)	
Denture use			
All-cause mortality			0.08
Without dentures	1.00 (1.00 to 1.00)	1.00 (1.00 to 1.00)	
With dentures	0.72 (0.62 to 0.84)	0.87 (0.75 to 1.01)	
Cardiovascular disease mortality			0.76
Without dentures	1.00 (1.00 to 1.00)	1.00 (1.00 to 1.00)	
With dentures	0.80 (0.58 to 1.10)	0.82 (0.59 to 1.13)	
Respiratory disease mortality			0.81
Without dentures	1.00 (1.00 to 1.00)	1.00 (1.00 to 1.00)	
With dentures	0.67 (0.37 to 1.22)	0.64 (0.43 to 0.95)	
Cancer mortality			0.60
Without dentures	1.00 (1.00 to 1.00)	1.00 (1.00 to 1.00)	
With dentures	0.88 (0.40 to 1.91)	0.94 (0.54 to 1.65)	
Other-cause mortality			0.11
Without dentures	1.00 (1.00 to 1.00)	1.00 (1.00 to 1.00)	
With dentures	0.69 (0.57 to 0.82)	0.88 (0.72 to 1.08)	

Figure S8 Association of the number of natural teeth and denture use with all-cause and cause-specific mortality stratified by sex. Multivariate models were adjusted for baseline age, marital status, education, residence, living arrangement, economic status, smoking status, drinking status, regular exercise, body mass index, hypertension, heart disease, diabetes mellitus, respiratory disease, cancer, fruit intake, vegetable intake, meat intake, fish intake, and egg intake, and further adjusted for denture use in the natural tooth and further adjusted for the number of natural teeth in the denture use model.HR, hazard ratio; CI, confidence interval.

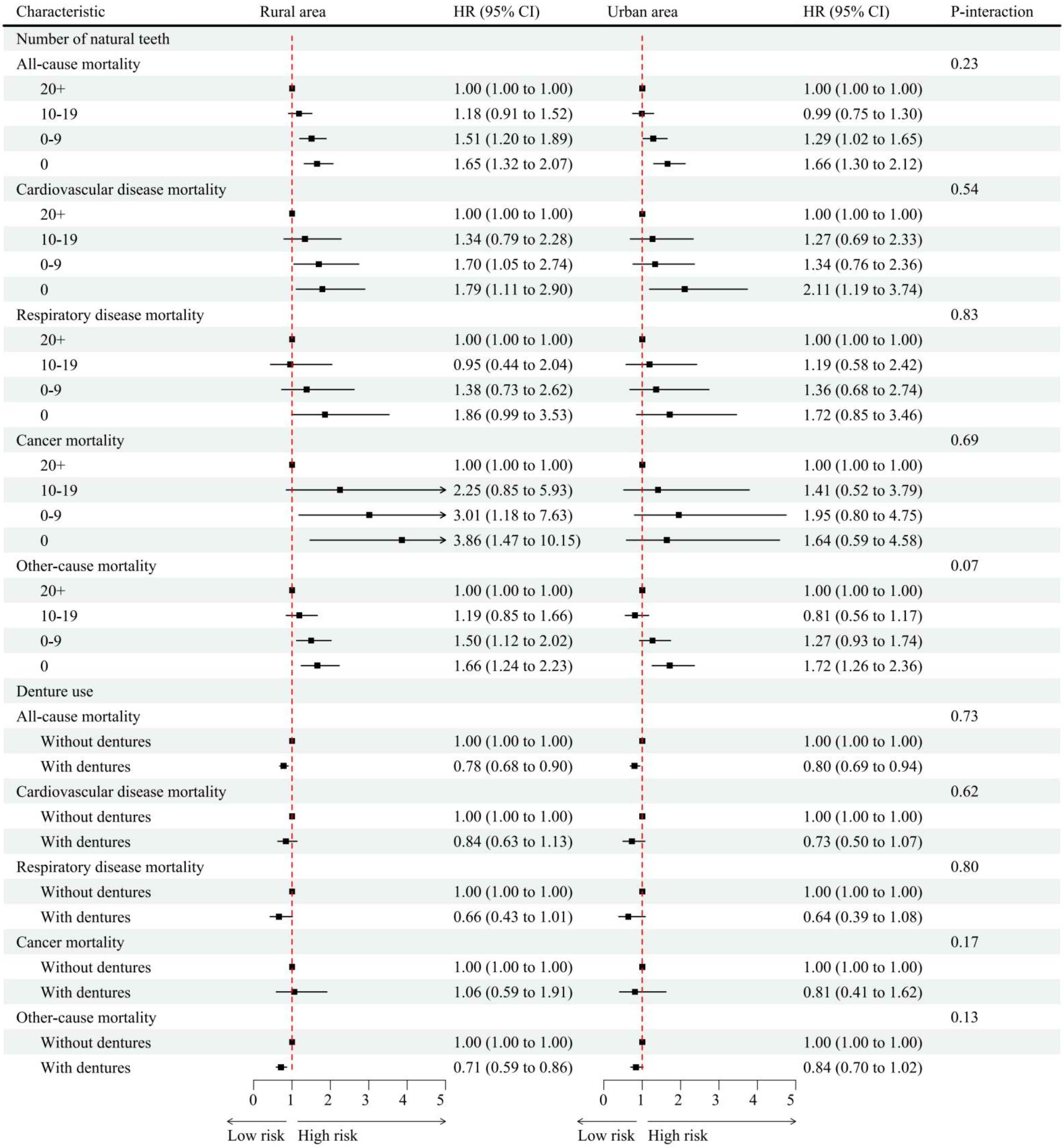

Characteristic	Rural area HR (95% CI)	Urban area HR (95% CI)	P-interaction
Number of natural teeth			
All-cause mortality			0.23
20+	1.00 (1.00 to 1.00)	1.00 (1.00 to 1.00)	
10-19	1.18 (0.91 to 1.52)	0.99 (0.75 to 1.30)	
0-9	1.51 (1.20 to 1.89)	1.29 (1.02 to 1.65)	
0	1.65 (1.32 to 2.07)	1.66 (1.30 to 2.12)	
Cardiovascular disease mortality			0.54
20+	1.00 (1.00 to 1.00)	1.00 (1.00 to 1.00)	
10-19	1.34 (0.79 to 2.28)	1.27 (0.69 to 2.33)	
0-9	1.70 (1.05 to 2.74)	1.34 (0.76 to 2.36)	
0	1.79 (1.11 to 2.90)	2.11 (1.19 to 3.74)	
Respiratory disease mortality			0.83
20+	1.00 (1.00 to 1.00)	1.00 (1.00 to 1.00)	
10-19	0.95 (0.44 to 2.04)	1.19 (0.58 to 2.42)	
0-9	1.38 (0.73 to 2.62)	1.36 (0.68 to 2.74)	
0	1.86 (0.99 to 3.53)	1.72 (0.85 to 3.46)	
Cancer mortality			0.69
20+	1.00 (1.00 to 1.00)	1.00 (1.00 to 1.00)	
10-19	2.25 (0.85 to 5.93)	1.41 (0.52 to 3.79)	
0-9	3.01 (1.18 to 7.63)	1.95 (0.80 to 4.75)	
0	3.86 (1.47 to 10.15)	1.64 (0.59 to 4.58)	
Other-cause mortality			0.07
20+	1.00 (1.00 to 1.00)	1.00 (1.00 to 1.00)	
10-19	1.19 (0.85 to 1.66)	0.81 (0.56 to 1.17)	
0-9	1.50 (1.12 to 2.02)	1.27 (0.93 to 1.74)	
0	1.66 (1.24 to 2.23)	1.72 (1.26 to 2.36)	
Denture use			
All-cause mortality			0.73
Without dentures	1.00 (1.00 to 1.00)	1.00 (1.00 to 1.00)	
With dentures	0.78 (0.68 to 0.90)	0.80 (0.69 to 0.94)	
Cardiovascular disease mortality			0.62
Without dentures	1.00 (1.00 to 1.00)	1.00 (1.00 to 1.00)	
With dentures	0.84 (0.63 to 1.13)	0.73 (0.50 to 1.07)	
Respiratory disease mortality			0.80
Without dentures	1.00 (1.00 to 1.00)	1.00 (1.00 to 1.00)	
With dentures	0.66 (0.43 to 1.01)	0.64 (0.39 to 1.08)	
Cancer mortality			0.17
Without dentures	1.00 (1.00 to 1.00)	1.00 (1.00 to 1.00)	
With dentures	1.06 (0.59 to 1.91)	0.81 (0.41 to 1.62)	
Other-cause mortality			0.13
Without dentures	1.00 (1.00 to 1.00)	1.00 (1.00 to 1.00)	
With dentures	0.71 (0.59 to 0.86)	0.84 (0.70 to 1.02)	

Figure S9 Association of the number of natural teeth and denture use with all-cause and cause-specific mortality stratified by place of residence. Multivariate models were adjusted for baseline age, marital status, education, living arrangement, economic status, smoking status, drinking status, regular exercise, body mass index, hypertension, heart disease, diabetes mellitus, respiratory disease, cancer, fruit intake, vegetable intake, meat intake, fish intake, and egg intake, and further adjusted for denture use in the natural tooth model and further adjusted for the number of natural teeth in the denture use model. HR, hazard ratio; CI, confidence interval.

Characteristic	HR (95% CI), below the median BMI	HR (95% CI), above the median BMI	P-interaction
**Number of natural teeth**			
All-cause mortality			0.87
20+	1.00 (1.00 to 1.00)	1.00 (1.00 to 1.00)	
10-19	1.05 (0.81 to 1.36)	1.14 (0.88 to 1.49)	
0-9	1.41 (1.12 to 1.78)	1.37 (1.09 to 1.73)	
0	1.67 (1.32 to 2.11)	1.56 (1.23 to 1.99)	
Cardiovascular disease mortality			0.51
20+	1.00 (1.00 to 1.00)	1.00 (1.00 to 1.00)	
10-19	1.04 (0.57 to 1.91)	1.46 (0.86 to 2.46)	
0-9	1.56 (0.91 to 2.67)	1.38 (0.84 to 2.25)	
0	1.75 (1.02 to 3.01)	1.68 (1.01 to 2.78)	
Respiratory disease mortality			0.06
20+	1.00 (1.00 to 1.00)	1.00 (1.00 to 1.00)	
10-19	0.47 (0.22 to 0.98)	1.73 (0.80 to 3.72)	
0-9	0.99 (0.54 to 1.80)	1.26 (0.59 to 2.69)	
0	1.06 (0.58 to 1.94)	2.22 (1.04 to 4.73)	
Cancer mortality			0.36
20+	1.00 (1.00 to 1.00)	1.00 (1.00 to 1.00)	
10-19	10.40 (1.23 to 85.64)	1.21 (0.56 to 2.65)	
0-9	14.53 (2.21 to 115.08)	1.29 (0.62 to 2.67)	
0	22.20 (3.36 to 180.35)	0.96 (0.41 to 2.25)	
Other-cause mortality			0.82
20+	1.00 (1.00 to 1.00)	1.00 (1.00 to 1.00)	
10-19	1.05 (0.75 to 1.45)	0.93 (0.64 to 1.36)	
0-9	1.37 (1.02 to 1.84)	1.46 (1.07 to 2.00)	
0	1.68 (1.25 to 2.25)	1.69 (1.23 to 2.33)	
**Denture use**			
All-cause mortality			0.79
Without dentures	1.00 (1.00 to 1.00)	1.00 (1.00 to 1.00)	
With dentures	0.72 (0.62 to 0.84)	0.79 (0.68 to 0.93)	
Cardiovascular disease mortality			0.80
Without dentures	1.00 (1.00 to 1.00)	1.00 (1.00 to 1.00)	
With dentures	0.80 (0.58 to 1.10)	0.81 (0.58 to 1.13)	
Respiratory disease mortality			0.42
Without dentures	1.00 (1.00 to 1.00)	1.00 (1.00 to 1.00)	
With dentures	0.67 (0.37 to 1.22)	0.56 (0.33 to 0.92)	
Cancer mortality			0.77
Without dentures	1.00 (1.00 to 1.00)	1.00 (1.00 to 1.00)	
With dentures	0.88 (0.40 to 1.91)	1.14 (0.63 to 2.04)	
Other-cause mortality			0.46
Without dentures	1.00 (1.00 to 1.00)	1.00 (1.00 to 1.00)	
With dentures	0.69 (0.57 to 0.82)	0.73 (0.59 to 0.90)	

Figure S10 Association of the number of natural teeth and denture use with all-cause and cause-specific mortality stratified by body mass index. Multivariate models were adjusted for baseline age, sex, marital status, education, residence, living arrangement, economic status, smoking status, drinking status, regular exercise, hypertension, heart disease, diabetes mellitus, respiratory disease, cancer, fruit intake, vegetable intake, meat intake, fish intake, and egg intake, and further adjusted for denture use in the natural tooth model and further adjusted for the number of natural teeth in the denture use model. HR, hazard ratio; CI, confidence interval; BMI, body mass index.

Characteristic	Economic dependence	HR (95% CI)	Economic independence	HR (95% CI)	P-interaction
Number of natural teeth					
All-cause mortality					0.51
20+		1.00 (1.00 to 1.00)		1.00 (1.00 to 1.00)	
10-19		1.08 (0.87 to 1.33)		1.12 (0.77 to 1.63)	
0-9		1.35 (1.12 to 1.62)		1.63 (1.15 to 2.31)	
0		1.61 (1.34 to 1.94)		1.71 (1.17 to 2.48)	
Cardiovascular disease mortality					0.95
20+		1.00 (1.00 to 1.00)		1.00 (1.00 to 1.00)	
10-19		1.23 (0.77 to 1.95)		1.39 (0.63 to 3.08)	
0-9		1.54 (1.03 to 2.30)		1.53 (0.68 to 3.45)	
0		1.85 (1.23 to 2.78)		1.84 (0.76 to 4.44)	
Respiratory disease mortality					0.89
20+		1.00 (1.00 to 1.00)		1.00 (1.00 to 1.00)	
10-19		1.02 (0.54 to 1.93)		0.96 (0.38 to 2.41)	
0-9		1.19 (0.69 to 2.07)		1.35 (0.56 to 3.27)	
0		1.72 (1.00 to 2.97)		1.71 (0.64 to 4.56)	
Cancer mortality					0.89
20+		1.00 (1.00 to 1.00)		1.00 (1.00 to 1.00)	
10-19		1.63 (0.59 to 4.48)		1.64 (0.65 to 4.17)	
0-9		1.83 (0.73 to 4.61)		2.11 (0.87 to 5.09)	
0		2.64 (1.01 to 6.88)		2.17 (0.77 to 6.15)	
Other-cause mortality					0.17
20+		1.00 (1.00 to 1.00)		1.00 (1.00 to 1.00)	
10-19		1.07 (0.82 to 1.41)		0.79 (0.44 to 1.42)	
0-9		1.37 (1.08 to 1.73)		1.54 (0.92 to 2.57)	
0		1.67 (1.32 to 2.11)		1.89 (1.11 to 3.22)	
Denture use					
All-cause mortality					0.55
Without dentures		1.00 (1.00 to 1.00)		1.00 (1.00 to 1.00)	
With dentures		0.78 (0.69 to 0.87)		0.86 (0.67 to 1.11)	
Cardiovascular disease mortality					0.42
Without dentures		1.00 (1.00 to 1.00)		1.00 (1.00 to 1.00)	
With dentures		0.77 (0.60 to 0.98)		0.94 (0.52 to 1.72)	
Respiratory disease mortality					0.41
Without dentures		1.00 (1.00 to 1.00)		1.00 (1.00 to 1.00)	
With dentures		0.60 (0.41 to 0.89)		0.86 (0.43 to 1.71)	
Cancer mortality					0.28
Without dentures		1.00 (1.00 to 1.00)		1.00 (1.00 to 1.00)	
With dentures		0.96 (0.53 to 1.74)		0.80 (0.39 to 1.64)	
Other-cause mortality					0.31
Without dentures		1.00 (1.00 to 1.00)		1.00 (1.00 to 1.00)	
With dentures		0.75 (0.65 to 0.87)		0.88 (0.62 to 1.26)	

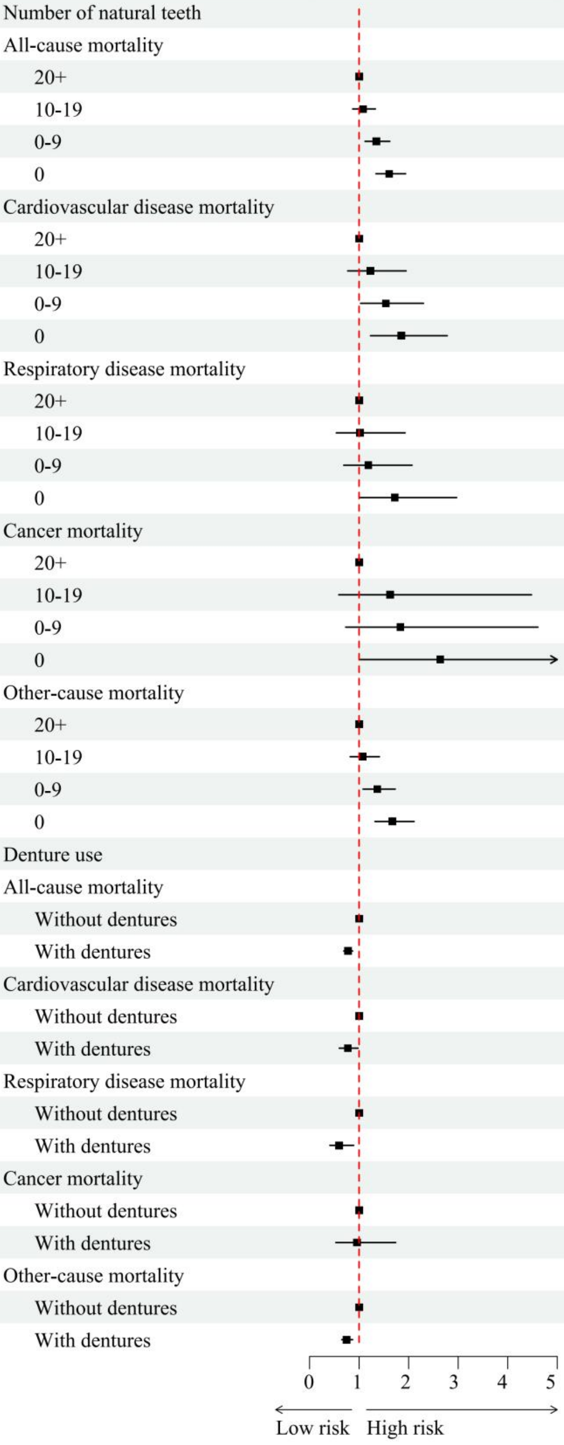

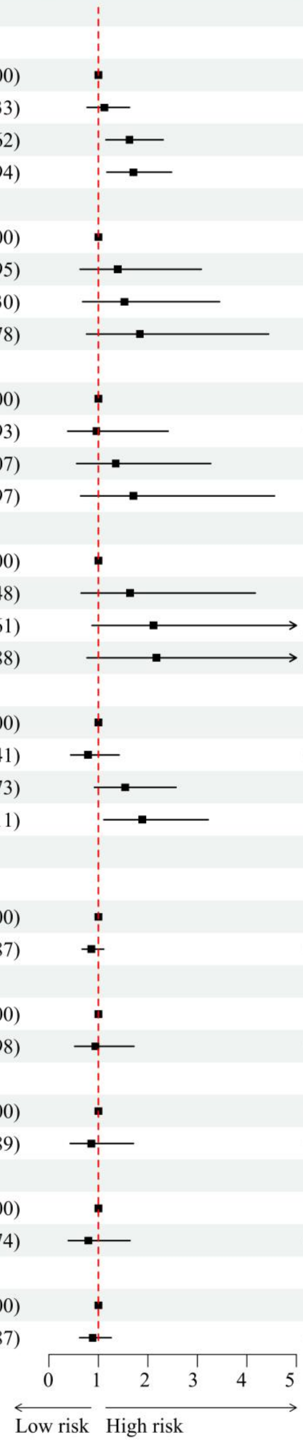

Figure S11 Association of the number of natural teeth and denture use with all-cause and cause-specific mortality stratified by economic status. Multivariate models were adjusted for baseline age, sex, marital status, education, residence, living arrangement, smoking status, drinking status, regular exercise, body mass index, hypertension, heart disease, diabetes mellitus, respiratory disease, cancer, fruit intake, vegetable intake, meat intake, fish intake, and egg intake, and further adjusted for denture use in the natural tooth and further adjusted for the number of natural teeth in the denture use model. HR, hazard ratio; CI, confidence interval.

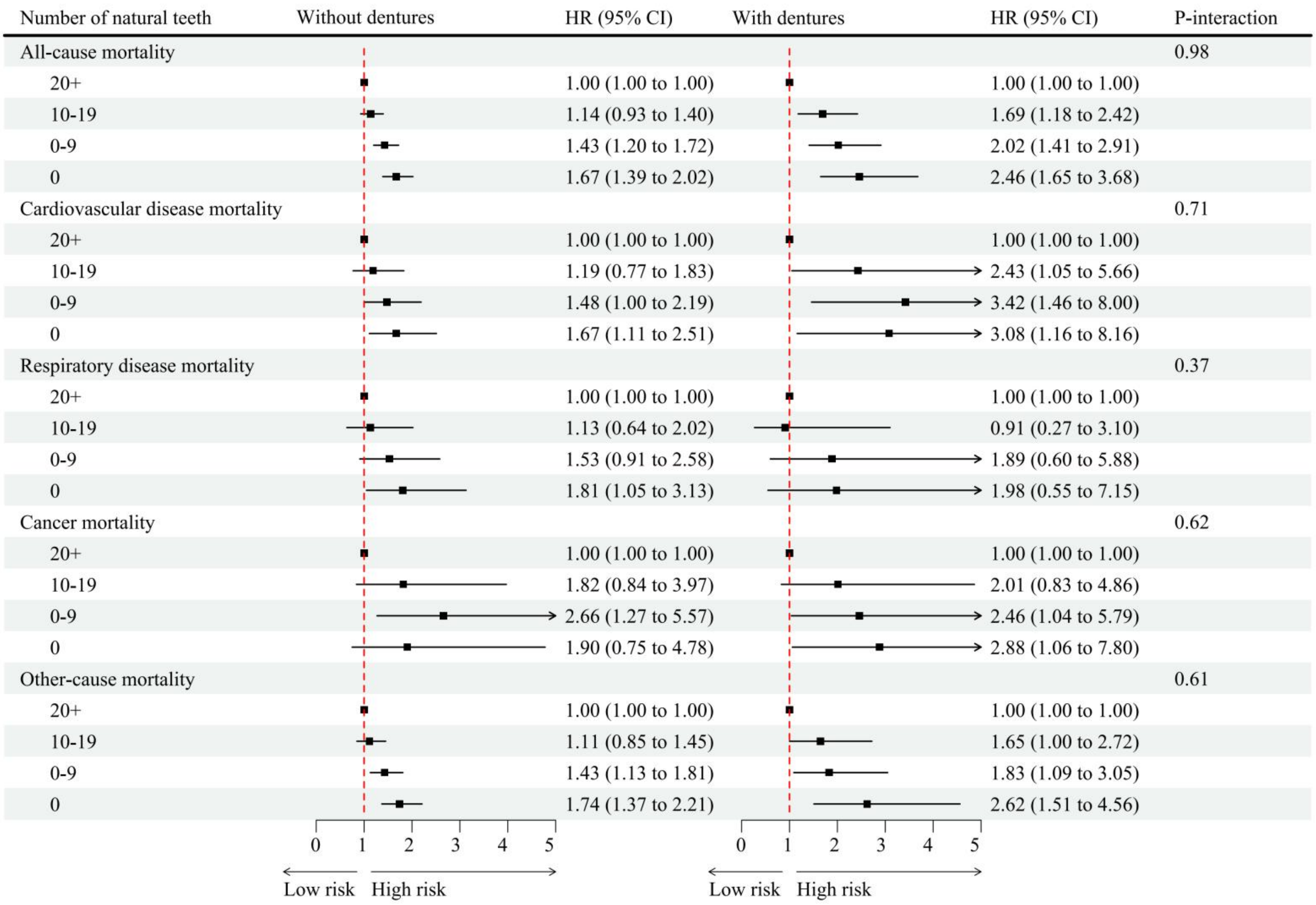

Figure S12 Association of the number of natural teeth with all-cause and cause-specific mortality stratified by denture use. Multivariate models were adjusted for baseline age, sex, marital status, education, residence, living arrangement, economic status, smoking status, drinking status, regular exercise, body mass index, hypertension, heart disease, diabetes mellitus, respiratory disease, cancer, fruit intake, vegetable intake, meat intake, fish intake, and egg intake. HR, hazard ratio; CI, confidence interval.

Table S1

Comparison of baseline characteristics between the overall participants and the included participants in the 2014 wave

Characteristics	Overall participants (n = 7,192)	Included participants (n = 5,403)	P value
Age (year), mean (SD)	85.32 (10.77)	85.44 (10.47)	0.51
Male, no. (%)	3,316 (46.1)	2,496 (46.2)	0.94
Married, no. (%)	2,885 (40.1)	2,184 (40.4)	0.74
Living with family member(s), no. (%)	5,639 (78.4)	4,232 (78.3)	0.93
Urban area, no.(%)	3,212 (44.7)	2,309 (42.7)	0.03
Smoking status, no. (%)			0.32
Never	5,060 (70.4)	3,858 (71.4)	
Current	1,201 (16.7)	850 (15.7)	
Former	931 (12.9)	695 (12.9)	
Drinking status, no. (%)			0.90
Never	5,408 (75.2)	4,071 (75.3)	
Current	1,094 (15.2)	807 (14.9)	
Former	690 (9.6)	525 (9.7)	
Regular exercise, no. (%)			0.07
Never	4,913 (68.3)	3,770 (69.8)	
Current	1,895 (26.3)	1,387 (25.7)	
Former	384 (5.3)	246 (4.6)	
Education (year), no. (%)			0.06
0	4,058 (56.4)	3,103 (57.4)	
1-6	2,279 (31.7)	1,731 (32.0)	
>6	855 (11.9)	569 (10.5)	
Economic independence, no. (%)	1,967 (27.3)	1,345 (24.9)	0.002
Number of natural teeth, no. (%)			0.37
0	2,510 (34.9)	1,929 (35.7)	
1-9	1,990 (27.7)	1,518 (28.1)	
10-19	1,208 (16.8)	908 (16.8)	
20+	1,484 (20.6)	1,048 (19.4)	
Denture use, no. (%)	2,529 (35.2)	1,896 (35.1)	0.95
BMI (kg/m^2), no. (%)			0.70
Underweight (<18.5)	1,359 (18.9)	1,048 (19.4)	
Normal (18.5-24)	4,004 (55.7)	3,024 (56.0)	
Overweight (24-28)	1,393 (19.4)	1,023 (18.9)	
Obese (≥28)	436 (6.1)	308 (5.7)	
Hypertension, no. (%)	2,430 (33.8)	1,758 (32.5)	0.15
Diabetes mellitus, no. (%)	427 (5.9)	281 (5.2)	0.08
Heart disease, no. (%)	999 (13.9)	665 (12.3)	0.01
Respiratory disease, no. (%)	855 (11.9)	591 (10.9)	0.10
Cancer, no. (%)	71 (1.0)	44 (0.8)	0.36
Fruit intake, no. (%)			0.86
Never	1,810 (25.2)	1,360 (25.2)	
Occasionally	2,449 (34.1)	1,863 (34.5)	
Almost daily	2,933 (40.8)	2,180 (40.3)	
Vegetable intake, no. (%)			0.62
Never	285 (4.0)	196 (3.6)	

Occasionally	595 (8.3)	452 (8.4)	
Almost daily	6,312 (87.8)	4,755 (88.0)	
Meat intake, no. (%)			0.42
Never	525 (7.3)	365 (6.8)	
Occasionally	3,925 (54.6)	2,993 (55.4)	
Almost daily	2,742 (38.1)	2,045 (37.8)	
Fish intake, no. (%)			0.73
Never	1,251 (17.4)	915 (16.9)	
Occasionally	5,376 (74.7)	4,072 (75.4)	
Almost daily	565 (7.9)	416 (7.7)	
Egg, no. (%)			0.66
Never	680 (9.5)	488 (9.0)	
Occasionally	4,362 (60.7)	3,310 (61.3)	
Almost daily	2,150 (29.9)	1,605 (29.7)	

Notes: Values are presented as number (%) or mean ± SD. Differences in characteristics were compared using the χ^2 test for categorical variables and the t-test for continuous variables. BMI, body mass index.

Table S2

The numbers (percentage) of the missing variables

Characteristics*	Number (%) with missing data
Living arrangement	40 (0.7)
Smoking status	15 (0.3)
Drinking status	31 (0.6)
Regular exercise	83 (1.5)
Education	42 (0.8)
Economic status	38 (0.7)
Marital status	63 (1.2)
Sleep time	36 (0.7)
Body mass index	446 (8.3)
Hypertension	97 (1.8)
Diabetes mellitus	119 (2.2)
Heart disease	112 (2.1)
Respiratory disease	116 (2.1)
Cancer	138 (2.6)
Fruit	14 (0.3)
Vegetable intake	13 (0.2)
Meat intake	44 (0.8)
Fish intake	45 (0.8)
Egg intake	44 (0.8)

*List of only the variables with missing data

Table S3

Sensitivity analyses for the association of the number of natural teeth and denture use with all-cause and cause-specific mortality risk

Characteristic	Removing participants with missing covariate data	Removing participants who had heart disease, respiratory disease, or cancer	Removing the participants who died within the first year of follow-up	After inverse probability weighting
	HR (95% CI)	HR (95% CI)	HR (95% CI)	HR (95% CI)
Number of natural teeth				
All-cause mortality				
20+	Reference	Reference	Reference	Reference
10-19	1.10 (0.89-1.34)	1.23 (0.99-1.53)	1.15 (0.94-1.42)	1.04 (0.67-1.60)
1-9	1.42 (1.18-1.70)	1.50 (1.24-1.83)	1.34 (1.11-1.62)	1.37 (0.96-1.95)
0	1.54 (1.28-1.86)	1.81 (1.48-2.20)	1.60 (1.32-1.94)	1.58 (1.11-2.26)
P value for trend [a]	<0.001	<0.001	<0.001	0.006
Number of teeth (continuous variable)	0.98 (0.97-0.99)	0.98 (0.97-0.98)	0.98 (0.97-0.99)	0.98 (0.97-0.99)
Cardiovascular disease mortality				
20+	Reference	Reference	Reference	Reference
10-19	1.43 (0.90-2.27)	1.34 (0.83-2.17)	1.65 (1.04-2.61)	1.62 (0.79-3.31)
1-9	1.74 (1.14-2.66)	1.64 (1.06-2.55)	1.88 (1.23-2.89)	2.02 (1.14-3.59)
0	1.75 (1.13-2.72)	1.85 (1.19-2.87)	1.80 (1.16-2.81)	2.15 (1.23-3.76)
P value for trend [a]	0.01	0.01	0.01	0.003
Number of teeth (continuous	0.98 (0.96-0.99)	0.98 (0.96-0.99)	0.98 (0.96-0.99)	0.97 (0.95-0.99)

variable)				
Respiratory disease mortality				
20+	Reference	Reference	Reference	Reference
10-19	0.96 (0.55-1.69)	1.35 (0.69-2.61)	0.94 (0.51-1.73)	0.72 (0.27-1.92)
1-9	1.02 (0.60-1.73)	1.57 (0.85-2.92)	1.26 (0.73-2.18)	0.71 (0.38-1.33)
0	1.39 (0.82-2.34)	1.87 (0.99-3.50)	1.67 (0.96-2.91)	1.15 (0.63-2.10)
P value for trend [a]	0.28	0.19	0.22	0.85
Number of teeth (continuous variable)	0.99 (0.97-1.01)	0.98 (0.96-1.00)	0.98 (0.96-1.00)	1.00 (0.98-1.02)
Cancer mortality				
20+	Reference	Reference	Reference	Reference
10-19	1.73 (0.83-3.63)	1.82 (0.86-3.83)	1.42 (0.69-2.95)	1.46 (0.62-3.45)
1-9	2.13 (1.06-4.27)	1.75 (0.84-3.63)	1.63 (0.81-3.28)	2.78 (1.21-6.37)
0	2.22 (1.04-4.71)	1.66 (0.76-3.66)	1.85 (0.88-3.91)	2.46 (1.10-5.50)
P value for trend [a]	0.01	0.19	0.07	0.006
Number of teeth (continuous variable)	0.97 (0.95-1.00)	0.98 (0.96-1.01)	0.98 (0.95-1.01)	0.96 (0.94-0.99)
Other cause mortality				
20+	Reference	Reference	Reference	Reference
10-19	1.15 (0.94-1.42)	1.16 (0.88-1.54)	1.04 (0.79-1.37)	1.08 (0.69-1.69)
1-9	1.34 (1.11-1.62)	1.50 (1.17-1.92)	1.21 (0.95-1.54)	1.53 (1.05-2.22)
0	1.60 (1.32-1.94)	1.92 (1.50-2.46)	1.60 (1.25-2.03)	1.88 (1.30-2.71)

P value for trend [a]	<0.001	<0.001	<0.001	<0.001
Number of teeth (continuous variable)	0.98 (0.97-0.99)	0.97 (0.96-0.98)	0.98 (0.97-0.99)	0.98 (0.96-0.99)
Denture use				
All-cause mortality				
Without dentures	Reference	Reference	Reference	Reference
With dentures	0.83 (0.74-0.94)	0.77 (0.68-0.86)	0.80 (0.71-0.90)	0.82 (0.71-0.94)
Cardiovascular disease mortality				
Without dentures	Reference	Reference	Reference	Reference
With dentures	0.88 (0.67-1.16)	0.79 (0.61-1.04)	0.90 (0.69-1.18)	0.78 (0.59-1.02)
Respiratory disease mortality				
Without dentures	Reference	Reference	Reference	Reference
With dentures	0.88 (0.61-1.26)	0.58 (0.38-0.90)	0.59 (0.40-0.88)	0.88 (0.58-1.33)
Cancer mortality				
Without dentures	Reference	Reference	Reference	Reference
With dentures	1.11 (0.69-1.78)	0.96 (0.57-1.62)	0.98 (0.59-1.63)	0.85 (0.55-1.33)
Other cause mortality				
Without dentures	Reference	Reference	Reference	Reference
With dentures	0.80 (0.71-0.90)	0.76 (0.65-0.88)	0.78 (0.67-0.91)	0.79 (0.67-0.92)

Notes: Model 1: adjusted for baseline age and sex. Model 2: further adjusted for marital status, education, residence, living arrangement, economic status, smoking status, drinking status, regular exercise, body mass index, hypertension, heart disease, diabetes mellitus, respiratory disease, cancer, fruit intake, vegetable intake, meat intake, fish intake, and egg intake, and further adjusted for denture use in the natural tooth model and further adjusted for the number of natural teeth in the denture use model. HR, hazard ratio; CI, confidence interval.
